# Estimates of disease burden caused by foodborne pathogens in contaminated dairy products in Rwanda

**DOI:** 10.1186/s12889-023-15204-x

**Published:** 2023-04-06

**Authors:** Amanda C. Sapp, Gabriela F. Nane, Mirna P. Amaya, Eugène Niyonzima, Jean Paul Hategekimana, John J. VanSickle, Ronald M. Gordon, Arie H. Havelaar

**Affiliations:** 1 Emerging Pathogens Institute, Global Food Systems Institute, Animal Sciences Department, University of Florida, Gainesville, FL USA; 2 Department of Applied Mathematics, Delft University of Technology, Delft, The Netherlands; 3 College of Agriculture, Animal Sciences and Veterinary Medicine, University of Rwanda, Musanze, Rwanda; 4 Food and Resource Economics Department, University of Florida, Gainesville, FL USA; 5 Abrams Public Health Center, Tucson, AZ USA

**Keywords:** Disease burden, Dairy, *Campylobacter*, *Salmonella*, *Cryptosporidium*, *Brucella*, *Mycobacterium bovis*, Rwanda, Incidence, Mortality, Disability-adjusted life years, Risk assessment

## Abstract

**Background:**

The Girinka program in Rwanda has contributed to an increase in milk production, as well as to reduced malnutrition and increased incomes. But dairy products can be hazardous to health, potentially transmitting diseases such as bovine brucellosis, tuberculosis, and cause diarrhea. We analyzed the burden of foodborne disease due to consumption of raw milk and other dairy products in Rwanda to support the development of policy options for the improvement of the quality and safety of milk.

**Methods:**

Disease burden data for five pathogens (*Campylobacter* spp., nontyphoidal *Salmonella enterica*, *Cryptosporidium* spp., *Brucella* spp., and *Mycobacterium bovis*) were extracted from the 2010 WHO Foodborne Disease Burden Epidemiology Reference Group (FERG) database and merged with data of the proportion of foodborne disease attributable to consuming dairy products from FERG and a separately published Structured Expert Elicitation study to generate estimates of the uncertainty distributions of the disease burden by Monte Carlo simulation.

**Results:**

According to WHO, the foodborne disease burden (all foods) of these five pathogens in Rwanda in 2010 was like or lower than in the Africa E subregion as defined by FERG. There were 57,500 illnesses occurring in Rwanda owing to consumption of dairy products, 55 deaths and 3,870 Disability Adjusted Life Years (DALYs) causing a cost-of-illness of $3.2 million. 44% of the burden (in DALYs) was attributed to drinking raw milk and sizeable proportions were also attributed to traditionally (16–23%) or industrially (6–22%) fermented milk. More recent data are not available, but the burden (in DALYs) of tuberculosis and diarrheal disease by all causes in Rwanda has declined between 2010 and 2019 by 33% and 46%, respectively.

**Conclusion:**

This is the first study examining the WHO estimates of the burden of foodborne disease on a national level in Rwanda. Transitioning from consuming raw to processed milk (fermented, heat treated or otherwise) may prevent a considerable disease burden and cost-of-illness, but the full benefits will only be achieved if there is a simultaneous improvement of pathogen inactivation during processing, and prevention of recontamination of processed products.

**Supplementary Information:**

The online version contains supplementary material available at 10.1186/s12889-023-15204-x.

## Background

The Government of Rwanda provides poor households with a heifer to increase access and consumption of milk, with the aims to reduce child malnutrition rates and increase household incomes of smallholder farmers. This program is locally known as Girinka (traditional greeting meaning “may you have cows”). Girinka program was launched in 2006 and is reported to have benefitted 248,566 families through to 2016, with an additional 101,434 targeted to achieve the program goal of 350,000 beneficiary families by 2017 [1]. These authors reported that Girinka contributed to an increase in agricultural production in Rwanda, especially milk production and (dairy) products, as well as reduced malnutrition and increased incomes. The Girinka program is ongoing, with 380,162 cows by distributed by 2020 [2]. The growth in milk production in Rwanda has indeed translated into increased milk consumption. The most recent published data indicate that the national milk production has increased from 444,337 MT in 2011 to 891,326 MT in 2020. The milk consumption per capita followed the same trend growing from 44.2 to 72 L per person annually during the same period [3].

In Rwanda, milk is typically produced by smallholders and is generally transported to milk collection centers (MCC) on bicycles or motorcycles without reliable refrigeration [4]. The MCCs are located mainly in the East, North and South of the country with the majority being in the East and North.

Recently there have been positive changes in the marketing of milk influenced by the Rwanda Dairy Competitiveness Program II (RDCP II) spearheaded by Land O’Lakes International Development. RDCP II collaborated with the Livestock Standards and Certification Services to promote the adoption of improved milk collection, handling, and transportation practices throughout the dairy sector. This resulted in a higher proportion of milk meeting the Rwanda Seal of Quality standards [5].

The increased production (and consumption) of milk, particularly by the households of the rural poor is noteworthy, and points to the success of Girinka. The Rwanda Dairy Development Project was launched in 2017 and was found to have a positive impact on income of dairy farmers, improved access to credit facilities and adopt better farming practices[6]. Habiyaremye et al. [7] have recently reviewed the subsequent programs to enhance the dairy sector in Rwanda and conclude that “while the dairy policies, programs, and regulations in Rwanda have paved the way for the development of the dairy sector and contributed to the provision and use of inputs and services, there are still challenges that need to be addressed.”. While the Rwanda Seal of Quality includes testing for general bacterial counts, there appears to have been little attention so far to minimize the contamination of milk with pathogenic bacteria. Milk, consumed in an unprocessed or inadequately processed form, can be hazardous to health, potentially transmitting diseases such as bovine brucellosis, tuberculosis, and diarrheal diseases from infected cows. Ndahetuye et al. [4] found high total bacterial counts and somatic cell counts in milk at the farm and MCC, indicating microbial contamination and poor udder health. Furthermore, they found that presence of the fecal indicator *Escherichia coli*, *Salmonella*, and *Brucella* antibodies in milk was common, but finding antibiotic residues in milk was uncommon.

The goal of the project “Rwanda Enhancement for Enabling Policy Support to the Dairy Sector” was to analyze and develop policy options to promote the stimulation of increased production and marketing of processed milk and milk products for both the domestic and export markets. This project is one of a suite of research activities in Rwanda, supported by the Feed the Future Innovation Lab for Livestock Systems, aimed at increasing the production and consumption of wholesome animal sourced food (ASF) by Rwandans [8].

To support the development of policy options for the improvement of the quality and safety of milk in Rwanda, we provide a baseline estimate of the disease burden attributable to different dairy products in Rwanda, based on data on the global burden of foodborne disease as presented by the World Health Organization (WHO). According to WHO, 420,000 deaths occurred globally among 600 million cases of foodborne disease (FBD) in 2010, causing a disease burden of 33 million Disability-Adjusted Life Years (DALY) [9]. It was estimated that Africa had the highest FBD burden of all regions analyzed (2,455 DALY per 100,000 population compared to a global average of 477 DALY per 100,000 population). The World Bank has estimated that the economic cost of FBD in low- and middle-income countries (LMIC) amounts to $95.2 billion per year nearly all of which is attributable to food bought in domestic markets, and the annual cost of treating foodborne illnesses is estimated at $15 billion [10]. While both assessments only capture part of the total burden and cost of FBD in LMIC, important public health and economic benefits could be achieved by improving food safety in LMIC.

Building on the WHO results, Li et al. [11] found that, for the sub-region AFRE in which Rwanda is located, the median burden of animal source foods was 459 (95% UI 294–625) DALY per 100,000 population. Of these, the disease burden for dairy was 58 (95% UI 35–99) DALY per 100,000 population.

This study is the first to extract data from the WHO estimates on a national basis in Rwanda to increase the meaningfulness of the results for national decision makers. Our objective was to estimate the disease burden associated with consuming raw milk in Rwanda, and to assess how much this burden could be reduced by increasing milk processing. To this end, we selected five pathogens that are commonly transmitted by dairy and combined FERG data on foodborne disease burden with attribution data to dairy, also from FERG, and more detailed attribution to different dairy products, obtained from a structured expert elicitation study specifically organized for this project.

## Methods

### Data

Disease burden data were extracted from the 2010 WHO Foodborne Disease Burden Epidemiology Reference Group (FERG) database for Rwanda. Methods are fully described in [12]. While all calculations were done at country level, FERG presented results on a global scale and for subregions. These are based on the official grouping of WHO Member States including the African Region (AFR). FERG further subdivided each region into subregions based on child and adult mortality as described by [13]: stratum A: very low child and adult mortality, stratum B: low child mortality and very low adult mortality, stratum C: low child mortality and high adult mortality, stratum D: high child and adult mortality, and stratum E: high child mortality and very high adult mortality. Rwanda was assigned to subregion AFRE. The reference year for the WHO estimates of the global burden of foodborne disease is 2010, more recent data are not available at this level of detail.

The WHO estimates apply a “top-down” approach. This means that the starting point for the analysis was data on occurrence of diseases that may be transmitted by dairy in Rwanda, based on routine data collection and analysis by WHO. For this study, data were available on incidence, mortality, and DALY of diarrheal diseases, brucellosis and tuberculosis. Additionally, these diseases were attributed to specific hazards. Diarrheal diseases were attributed to many viruses, bacteria and protozoa. Such data are incomplete for most if not all countries in the world, and the estimates were based on systematic literature reviews and stratified by geographic region as appropriate. For the current analysis, we extracted data for diarrheal pathogens of which cattle are one of the reservoirs, i.e., *Campylobacter* spp., nontyphoidal *Salmonella enterica* and *Cryptosporidium* spp. Brucellosis is fully attributed to *Brucella* spp. Finally, the proportion of tuberculosis cases and deaths that was attributable to *Mycobacterium bovis* was estimated [14].

Bovine tuberculosis due to infection with *M. bovis* is an endemic disease in Rwanda [15] and is fully attributed to contaminated dairy products. The other hazards can also be transmitted by dairy but also through other pathways (e.g., water, animal-person transmission). Therefore, additionally, the proportion of all diseases attributable to food was estimated [8]. As a final step in the WHO estimates, the foodborne disease burden was attributed to food groups as relevant, and we used the proportion attributable to dairy for our analysis [16].

Disease burden estimates are expressed as incidence, mortality, and DALY at the population level and as rates per 100,000 population. Data for five zoonotic hazards transmissible by dairy (*Campylobacter* spp. (CAMP), nontyphoidal *Salmonella enterica* (NTS), *Cryptosporidium* spp. (CRYP), *Brucella* spp. (BRUC), and *Mycobacterium bovis* (MBOV) were filtered from the 31 hazards in the FERG database for the total population, children under 5 years of age and older children (5–19 years of age) and adults (> 19 years of age) combined.

Estimates of the proportion of foodborne disease attributable to dairy by the selected hazards were based on FERG results for the AFRE subregion as described by [17]. Attribution of the burden of the dairy food *group* to different dairy *types* (milk from cattle and milk from other animals) and to different cattle milk *products* (raw milk, milk fermented by traditional processes, milk fermented by industrial processes, heat treated milk and other milk products) were based on a structured expert judgment (SEJ) analysis organized specifically for this study. This analysis, also applied to food attribution questions in two other African countries (Ethiopia and Burkina Faso) is described in detail elsewhere [18]. Briefly, the SEJ analysis was done according to Cooke’s Classical Model [19, 20].

The Classical Model elicits, for quantities of interest, uncertain estimates in the form of distribution quantiles, typically the 5%, 50% (or the median) and the 95% quantiles. The three elicited estimates are regarded as lower, best and upper uncertainty estimates. Nonparametric distributions are then constructed for each expert, and experts’ distributions are aggregated using performance-based weights. The weights are obtained from experts’ performance in assessing uncertainty, evaluated with respect to statistical accuracy and informativeness. Statistical accurate assessments depict that true values for the calibration questions occur with expected frequencies, i.e., 5% of the true values are lower than expert’s 5% quantiles, 45% of the true values lie between expert’s 5% and 50% quantiles, etc. Differences between this expected and the actual realized frequency yield the calibration score or the statistical accuracy. Intuitively, informativeness reveals how concentrated experts’ assessments are.

The term “expert” as used in this context designated a person whose present or past field contained the subject study matter, and who was regarded by others as being one who is knowledgeable about the subject. More specifically, experts sought for this study had a diversity of backgrounds with experience in one or more of the following domains: diarrheal diseases, zoonoses, microbial food safety, water and sanitation or veterinary public health. Experts from Rwanda with a working knowledge of the safety of dairy in Rwanda were identified by the study team and invited to participate in the study by email. Experts who were willing to participate completed an online questionnaire about their working knowledge and experience, as well as uploaded their CV. They were also invited to suggest additional experts. Experts were then vetted by two senior investigators (RMG and AHH) based on their CV and included if they had as expertise one or more areas of diarrheal disease in humans, zoonoses, microbial food safety, or veterinary public health. Experts were not paid for their services.

A background document summarizing current surveillance data, where available, and relevant research findings for each pathogen was provided to the experts, as well as a training video on providing quantitative estimates under uncertainty (https://www.youtube.com/watch?v=kQHoldpo4tA&t=194s; duration 25 min) which was completed by all experts before being interviewed. The video provided details about the process of quantifying uncertainty, the method used to validate and aggregate expert opinion and the steps of the online elicitation. Individual interviews were held via the Zoom platform and had a duration of 60–90 min. Interviews started with an introduction to the study and an interactive discussion on the concept of uncertainty. Next, experts provided their assessments for calibration questions and shared these calibration results through email before the interview concluded. Within the Classical Model, calibration questions are used to assess the performance of experts’ assessments to quantify uncertainty and, in turn, to determine the performance-based weights used to aggregate experts’ distributions. The weights are derived from two performance measures, statistical accuracy and informativeness [19, 20] which are used to objectively quantify the ability of an expert to provide valid estimates of uncertain quantities. Next, interviewers introduced the target questions and walked through an example. Target questions are the actual unknown quantities for which the research team seeks estimates. After the interview, experts were allotted two weeks to research, consult the background document, and complete answers to the target questions, which they then returned to the interviewer. All data collection was done through custom-made Excel files (Microsoft, Redmond, WA). Data analysis was performed according to standard methodology of the Classical Model using the Excalibur software [21], specifically developed to analyze expert data. This analysis provided estimates of the uncertainty distribution of each target question individually. The target questions were designed to be comprehensive and mutually exclusive at the level of both food types and food products as defined below. Experts were asked to attribute cases of illness due to the five hazards in the dairy food *group* to different dairy *types* (milk from cattle and milk from other animals) and subsequently to attribute the illnesses attributed to cattle milk to five different cattle milk *products*: consumed raw, traditionally fermented (e.g., ikivuguto), industrially fermented, heat treated, or other products. Attribution to food types and food products was designed to be comprehensive and mutually exclusive. For example, for milk from cattle, disease transmission by the five different food products together should cover exactly 100% of all disease cases. Therefore, as a last step, the univariate uncertainty distributions resulting from the SEJ analysis were merged in a normalized joint uncertainty distribution for all food types or products. The normalization procedure was undertaken to ensure that average estimates of food products and food types summed to average estimates of food types and food groups, respectively. The normalization procedure is detailed in [22].

Disease burden data from FERG and attribution results were merged using Monte Carlo simulation to generate estimates of the uncertainty distributions of the disease burden per food type or food product for all hazards individually and summed over all hazards. These estimates were produced for the total population, children under 5 years of age and older children and adults. We assume, in agreement with FERG, that mortality and DALY metrics are proportional to the incidence of disease and use the same attribution estimated for all burden metrics.

All data extraction, manipulation, tables, plots and statistical testing were done in the R statistical software version 3.6.0 [23].

## Results

### Foodborne disease burden

Table 1 shows a comparison of global, sub-regional (Rwanda is in subregion AFRE) and country level data for Rwanda for the standardized burden of foodborne disease (DALY per 100,000 population) by the five hazards included in this study. FERG reports medians as point estimates for the uncertainty distribution. In this work, we have chosen to report means because, in contrast to medians, these are additive: the sum of means is the same as the mean of sums. For Rwanda, we report both medians and means in Table 1. Mean and median disease burden are very similar for all hazards except for BRUC, where the mean is approximately 10 times higher than the median, indicating a highly skewed distribution. The burden of CAMP in AFRE is approximately twice as high as the global average, and at about the same level in Rwanda as in the subregion. The burden of NTS in AFRE is approximately three times higher than the global average, and slightly lower than this average in Rwanda. The burden of CRYP in AFRE is approximately three times higher than the global average, and about the same as this average in Rwanda. The median burden of BRUC in AFRE is approximately six times lower than the global average, and about the same level in Rwanda (brucellosis is particularly a problem in the Eastern Mediterranean region). The burden of MBOV in AFRE is approximately four times higher in AFRE than the global average, but the burden in Rwanda is only slightly higher than the global average.

**Table 1:** Foodborne Disability-Adjusted Life Years (DALY) per 100,000 population for dairy-associated hazards for the global population, subregion AFRE* and Rwanda according to WHO FERG

Hazard	Global	AFRE	Rwanda
*Campylobacter* spp.	31 (22–46)^^^	70 (33–117)	71/73 (33–118)^&^
Non-typhoidal *S. enterica*	59 (36–91)	193 (44–336)	121/122 (27–211)
*Cryptosporidium* spp.	4 (2–11)	12 (0–45)	12/15 (0–46)
*Brucella* spp.	2 (0.6–42)	0.3 (0.007-18)	0.3/2.8 (0.002-20)
*Mycobacterium bovis*	9 (7–12)	34 (21–48)	13/13 (6–23)

^*^Subregion AFRE includes Botswana; Burundi; Central African Republic; Congo; Côte d’Ivoire; Democratic Republic of the Congo; Eritrea; Ethiopia; Kenya; Lesotho; Malawi; Mozambique; Namibia; Rwanda; South Africa; Swaziland; Uganda; United Republic of Tanzania; Zambia; Zimbabwe

^^^ Median (95% uncertainty interval)

^&^ Median/mean (95% uncertainty interval)

Table 2 compares different metrics of the burden of foodborne disease by the five selected hazards in Rwanda. CAMP incidence is more than twofold higher than NTS incidence and much higher than for CRYP, BRUC, and MBOV. The mortality incidence of NTS is more than twofold higher than of CAMP, and much higher than of CRYP, BRUC and MBOV. This results in a considerably higher number of life years lost due to NTS, and an overall higher foodborne disease burden as measured by DALY.

**Table 2:** Foodborne disease burden for five dairy-associated hazards in Rwanda (total population) according to WHO FERG [5]

Hazard	Incidence (x 1,000)	Incidence per 100,000	Mortality	Mortality per 100,000	DALY (x 1000)	DALY per 100,000
*Campylobacter* spp.	304 (33–939)^^^	2,800 (310-8,660)	85 (39–141)	0.79 (0.36–1.30)	7.87 (3.60–12.8)	72.6 (33.2–118)
Non-typhoidal *S. enterica*	117 (11–348)	1,080 (100-3,210)	175 (39–300)	1.61 (0.36–2.76)	13.2 (2.9–22.9)	122 (27–211)
*Cryptosporidium* spp.	28.5 (0.0 -101)	263 (0-936)	19.7 (0.00-59.1)	0.18 (0.00-0.55)	1.64 (0.00-4.96)	15.1 (0.0-45.8)
*Brucella* spp.	0.96 (0.00-7.22)	8.89 (0.01-67.0)	4.84 (0.00–35.0)	0.05 (0.00-0.32)	0.31 (0.00-2.21)	2.84 (0.00-20.4)
*Mycobacterium bovis*	0.27 (0.17–0.35)	2.46 (1.60–3.20)	24.8 (10.9–45.3)	0.23 (0.10–0.42)	1.41 (0.66–2.51)	13.0 (6.1–23.2)
Total burden	451 (89 − 1,200)	4,160 (820 − 10,700)	310 (128–493)	2.86 (1.18–4.55)	24.4 (10.0-39.2)	225 (92–362)

^^^ Mean (95% uncertainty interval)

While 15% of the population of Rwanda are children under the age of 5 [20], they bear 56% of the total burden of foodborne DALY (Table 3). The age distribution differs markedly between pathogens with children under the age of 5 bearing approximately 70% of the burden of CAMP and CRYP and approximately half of the burden of NTS, while their share in the burden of BRUC and MBOV is negligible.

**Table 3:** Proportion of foodborne disease burden borne by children under five years of age for five dairy-associated hazards in Rwanda, 2010 according to WHO FERG [5]

Hazard	Incidence	Mortality	DALY per year	
*Campylobacter* spp.	0.67 (0.06–0.98) ^^^	0.66 0.51–0.78)	0.70 (0.59–0.82)	
Non-typhoidal *S. enterica*	0.42 (0.02–0.90)	0.42 (0.31–0.54)	0.51 (0.39–0.61)	
*Cryptosporidium* spp.	0.81 (0.36–0.99)	0.63 (0.40–0.80)	0.69 (0.47–0.84)	
*Brucella* spp.	0.01 (0.01–0.01)	0.01 (0.01–0.01)	0.02 (0.02–0.02)	
*Mycobacterium bovis*	0.01 (0.01–0.01)	0.01 (0.01–0.01)	0.01 (0.01–0.01)	
Total foodborne burden	0.66 (0.21–0.95)	0.47 (0.36–0.56)	0.56 (0.45–0.65)	

^^^ Mean (95% uncertainty interval)

### Attribution of disease burden to specific foods

Table 4 shows the attribution estimates for foodborne disease burden due to the five selected hazards in the AFRE subregion to dairy products. Attribution of foodborne disease to dairy is based on FERG [11], attribution to dairy types and attribution to dairy products was elicited during the SEJ analysis [16]. Note that according to FERG, transmission of MBOV was 100% by dairy because this organism is only shed in milk. Attribution to dairy was also relatively high for BRUC, and much lower for the other hazards. For all hazards, the majority of dairy associated cases (89–94%) were attributed to milk from cattle. Only one-third of the cases of dairy-associated cases of CAMP and just over 40% of cases of NTS and CRYP were attributed to raw cattle milk, while 41% of cases of CAMP and just over one-third of cases of NTS and CRYP were attributed to traditionally or industrially fermented dairy products. In contrast, just under two-thirds of cases of BRUC and MBOV were attributed to raw milk from cattle with 16–19% attributed to traditionally fermented milk products.

**Table 4:** Attribution of foodborne disease due to five dairy-associated hazards in Rwanda to the dairy food group, dairy types and products. Table 4A provides estimates of the proportion of foodborne disease that is attributed to the dairy food group, based on WHO FERG [11]. Table 4B provides unconditional probabilities of attributing the burden of a foodborne pathogen to dairy types and products, based on [16]. For example, 89% (represented in tables as a proportion of 0.89) of the dairy-associated burden of *Campylobacter* spp. is attributed to milk from cattle. Food types are indicated in italics. Of the 89% attributed to milk from cattle, 33% is attributed to consuming raw cattle milk, while other food products make up for the remaining 56%. Food products are indicated in plain text A. Dairy food group

Pathogen	Attribution estimates	
*Campylobacter* spp.	0.15 (0.01–0.22)^^^	
Non-typhoidal *Salmonella enterica*	0.07 (0.01–0.19)	
*Cryptosporidium* spp.	0.11 (0.00-0.46)	
*Brucella* spp.	0.68 (0.50–0.86)	
*Mycobacterium bovis*	1.00 (1.00–1.00)	

**Table:** B. Dairy food types and food products

Food groups^*^	Food types	Food products	Attribution estimates
*Campylobacter* spp.			
	*Milk from cattle*		*0.89 (0.50-1.00)* ^^^
		Raw	0.33 (0.00-0.68)
		Fermented traditional	0.19 (0.00-0.56)
		Fermented industrial	0.22 (0.00-0.59)
		Heat treated	0.07 (0.00-0.45)
		Other products	0.08 (0.00-0.43)
	*Milk from other animal species*		*0.11 (0.00-0.50)*
Non-typhoidal *Salmonella enterica*			
	*Milk from cattle*		*0.94 (0.50-1.00)*
		Raw	0.46 (0.12–0.74)
		Fermented traditional	0.22 (0.01–0.49)
		Fermented industrial	0.15 (0.01–0.42)
		Heat treated	0.06 (0.00-0.43)
		Other products	0.05 (0.00-0.35)
	*Milk from other animal species*		*0.06 (0.00-0.50)*
*Cryptosporidium* spp.			
	*Milk from cattle*		*0.89 (0.50-1.00)*
		Raw	0.43 (0.05–0.79)
		Fermented traditional	0.23 (0.00-0.57)
		Fermented industrial	0.10 (0.00-0.38)
		Heat treated	0.05 (0.00-0.41)
		Other products	0.08 (0.00-0.34)
	*Milk from other animal species*		*0.11 (0.00-0.50)*
*Brucella* spp.			
	*Milk from cattle*		*0.93 (0.49-1.00)*
		Raw	0.61 (0.19–0.90)
		Fermented traditional	0.19 (0.00-0.48)
		Fermented industrial	0.06 (0.00-0.32)
		Heat treated	0.04 (0.00-0.42)
		Other products	0.03 (0.00-0.19)
	*Milk from other animal species*		*0.07 (0.00-0.51)*
*Mycobacterium bovis*			
	*Milk from cattle*		*0.94 (0.50-1.00)*
		Raw	0.63 (0.19–0.91)
		Fermented traditional	0.16 (0.01–0.50)
		Fermented industrial	0.06 (0.00-0.34)
		Heat treated	0.05 (0.00-0.43)
		Other products	0.04 (0.00-0.20)
	*Milk from other animal species*		*0.06 (0.50-1.00)*

^^^ Mean (95% uncertainty interval)

**Fig. 1:**
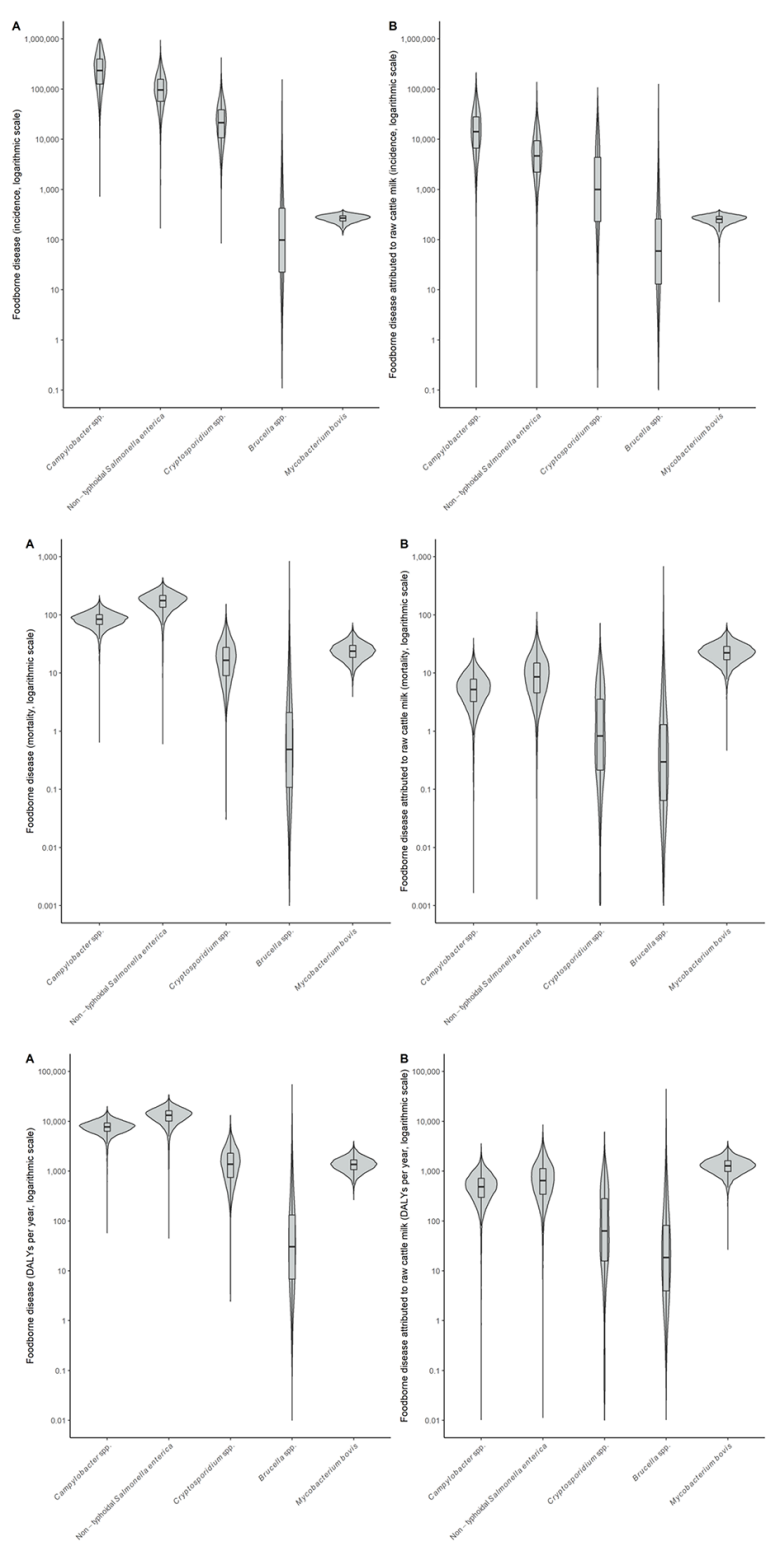
Uncertainty distributions of foodborne disease burden (panels A, left) and disease burden associated with consuming raw milk (panels B, right) in Rwanda, reporting incidence (top row), mortality (middle row) and DALY (bottom row) Violin plots display two mirror images of the probability density function of a distribution, smoothed by a kernel density estimator. Embedded in the violin plot is a boxplot, showing lines for the median, boxes for the interquartile range and whiskers for the 95% uncertainty intervals. Values beyond the 95% UI have been omitted. Note the y-axis is on a logarithmic scale

Table 5 shows the burden of dairy in Rwanda for the total population due to the five selected hazards. Similar data for children under 5 years of age and for children over 5 years of age and adults as well as a breakdown of DALY in Years of Life Lost and Years Lived with Disability are presented in S1-S5 Tables. Annually, there are 57,500 cases of illness due to consumption of contaminated dairy, of which 44,600 (76%) are caused by CAMP. These illnesses cause 55 deaths per year, mainly associated with MBOV (25 cases, 45%). The total disease burden of consuming contaminated dairy in Rwanda is about 3,870 DALY per year, of which 1,150 due to CAMP, 850 due to NTS and 1,410 due to MBOV (Fig. 1). The burden of CRYP and BRUC is lower. Of this burden, 13,300 illnesses, 26 deaths and 1,700 DALY is attributed to drinking raw cattle milk. A sizeable proportion of the burden was also attributed to traditionally (16–23%) or industrially (6–22%) fermented milk (S1-S5 Tables).

**Table 5:** Disease burden associated with dairy consumption in Rwanda, 2010 (total population) A. All dairy groups

Hazard	Incidence (x 1,000)	Incidence per 100,000	Mortality	Mortality per 100,000	DALY (x 1000)	DALY per 100,000
*Campylobacter* spp.	44.6 (0.9–172)^^^	411 (8 − 1,590)	12.5 (0.4–31.8)	0.12 (0.00-0.29)	1.15 (0.41–2.91)	10.6 (0.4–26.9)
Non-typhoidal *S. enterica*	7.56 (0.00-315)	69.7 (0.0-291)	11.3 (0.0–37.0)	0.11 (0.00-0.34)	0.85 (0.00-2.78)	7.87 (0.00-25.7)
*Cryptosporidium* spp.	4.47 (0.00-29.8)	41.2 (0.0-275)	3.06 (0.00–18.0)	0.03 (0.00-0.17)	0.25 (0.00-1.50)	2.35 (0.00-13.9)
*Brucella* spp.	0.65 (0.00-4.48)	6.02 (0.00-41.3)	3.28 (0.00-23.3)	0.03 (0.00-0.22)	0.21 (0.00-1.46)	1.92 (0.00-13.5)
*Mycobacterium bovis*	0.27 (0.17–0.35)	2.46 (1.60–3.20)	24.8 (10.9–45.3)	0.23 (0.10–0.42)	1.41 (0.66–2.51)	13.0 (6.1–23.2)
Total burden	57.5 (6.6–190)	531 (61 − 1,750)	54.9 (25.8–99.0)	0.51 (0.24–0.91)	3.87 (1.73–7.16)	35.7 (16.0–66.0)

^^^ Mean (95% uncertainty interval)

**Table:** B. Raw cattle milk consumption

Hazard	Incidence (x 1,000)	Incidence per 100,000	Mortality	Mortality per 100,000	DALY (x 1000)	DALY per 100,000
*Campylobacter* spp.	7.33 (0.00-33.5)^^^	67.6 (0.0-309)	2.04 (0.00-6.84)	0.02 (0.00-0.06)	0.18 (0.00-0.63)	1.74 (0.00-5.81)
Non-typhoidal *S. enterica*	3.48 (0.00-15.5)	32.1 (0.0-143)	5.21 (0.00–19.0)	0.05 (0.00-0.18)	0.39 (0.00-1.42)	3.62 (0.00-13.2)
*Cryptosporidium* spp.	1.92 (0.00-13.4)	17.7 (0.0-124)	1.32 (0.00-8.50)	0.01 (0.00-0.08)	0.11 (0.00-0.71)	1.01 (0.00-6.55)
*Brucella* spp.	0.38 (0.00-2.87)	3.52 (0.00-26.5)	1.92 (0.00-14.4)	0.02 (0.00-0.13)	0.12 (0.00-0.91)	1.13 (0.00-8.41)
*Mycobacterium bovis*	0.17 (0.05–0.28)	1.56 (0.43–2.59)	15.7 (3.5–33.0)	0.15 (0.03–0.30)	0.89 (0.20–1.84)	8.23 (1.81-17.0)
Total burden	13.3 (1.2–46)	123 (11–425)	26.2 (6.6–56.1)	0.24 (0.06–0.52)	1.70 (0.44–3.73)	15.7 (4.1–34.4)

^^^ Mean (95% uncertainty interval)

## Discussion

This study has presented the first opportunity to explore the WHO global estimates of foodborne disease on a country-specific basis to support national food safety decision making. Even though the data refer to the reference year 2010, they are still useful to support policy making when considered in the context of limited available data on trends in disease burden in Rwanda. According to data from the Global Burden of Disease study published by the Institute for Health Metrics and Evaluation [24], the disease burden (DALY per 100,000) for tuberculosis by all causes decreased in Rwanda by 33% (95% confidence interval 19–46%) and for diarrhea by all causes by 46% (24–64%) between 2010 and 2019. No specific data on changes in the burden of *Brucella* spp., *M. bovis* or any of the diarrheal pathogens considered in this study are available. These changes are mainly driven by a decrease in the number of deaths. It is therefore likely that the decrease in disease burden can be attributed to a more effective health care system rather than to improved safety of dairy products, which would have resulted in a reduced incidence.

Using World Bank methodology [10], equating 1 DALY per year to the Gross National Income per capita (USD 820 in 2019 for Rwanda: https://data.worldbank.org/indicator/NY.GNP.PCAP.CD, accessed March 24, 2021) results in a cost of $USD 3.2 million per year associated with consuming dairy. Of these costs, USD 1.4 million could be averted by replacing raw milk consumption by effectively processed products.

The FERG data, combined with new attribution estimates generated for this study and published separately [18], suggest a considerable burden of campylobacteriosis, salmonellosis and tuberculosis from dairy in Rwanda. In comparison, the burden of cryptosporidiosis and brucellosis was relatively small. In 2010, consuming contaminated dairy products in Rwanda resulted in 57,500 cases of illness, 55 deaths, a burden of 3,870 DALY and costs of USD 3.4 million. A large share of this burden was borne by children under 5 years of age. There are different ways to prevent or reduce this burden. About two-thirds of the total burden was estimated to be due to zoonotic fecal pathogens (*Campylobacter* spp. and non-typhoidal *Salmonella enterica*). This suggests that improved hygiene during milking and milk collection would be an effective intervention method. Approximately one-third of the burden was estimated to be due to bovine tuberculosis, which is a systemic infection in the cow and would still contaminate the milk even if milking and milk collection would be done hygienically. Additional interventions to reduce infection of cattle would be necessary. Alternatively, milk processing using a proper heating step would kill all pathogens present in the raw milk, thus providing a single intervention to reduce the full disease burden. Heat treatment is an established technology to reduce the risk of human infections attributable to the consumption of contaminated foods. However, attribution of *Brucella* spp. and *Mycobacterium bov*is to traditionally fermented milk products and to a lesser extent to other dairy products suggests experts considered failure of (particularly traditional) processing technologies to fully inactivate pathogens. The even higher attribution of *Campylobacter* spp., non-typhoidal *Salmonella enterica* and *Cryptosporidium* spp. to processed milk suggests that for these pathogens, they additionally considered recontamination after processing was likely to happen. Recontamination is a challenge to the dairy industry even in high-technology environments [25] and even more in low-income settings [26].These considerations limit the benefits of policy changes intended to transitioning from raw to processed milk consumption unless improved controls of processing technologies and prevention of recontamination of processed products are implemented concurrently.

Owing to data limitations, the uncertainty margins in the estimates of the disease burden are considerable and span several orders of magnitude (Fig. 1). The uncertainty is smallest for MBOV, followed by CAMP and NTS with distributions showing longer left tails. Uncertainties are larger for CRYP and particularly BRUC, for which the 95% uncertainty intervals span six orders of magnitude. This uncertainty needs to be considered when using these results for further decision making, e.g., by cost-benefit calculations. This would require collecting more quantitative information on the epidemiology of foodborne disease in Rwanda and the structure of the dairy value chain in Rwanda as well as building microbial risk assessment and economic models.

## Conclusion

We demonstrate a considerable disease burden associated with consuming dairy products in Rwanda. 23% of all cases, 48% of deaths and 44% of Disability-Adjusted Life Years was associated with drinking raw milk,. Discouraging consumption of raw milk may reduce this burden but should be accompanied by interventions to increase the process control of heat treatment and prevent recontamination of processed dairy products as a significant burden was also attributed to industrially processed dairy products (12% of cases, 9% of deaths and 11% of DALYs).

## Electronic supplementary material

Below is the link to the electronic supplementary material.

Supplementary Material 1

Supplementary Material 2

Supplementary Material 3

Supplementary Material 4

Supplementary Material 5

## Data Availability

The datasets generated and/or analyzed during the current study are available in the Harvard Dataverse repository 10.7910/DVN/53SEJY.
