# Supplementary material for: Estimates of disease burden caused by foodborne pathogens in contaminated dairy products in Rwanda
Source: BMC Public Health. 2023 Apr 6;23:657. doi: 10.1186/s12889-023-15204-x (PMC10077627; doi:10.1186/s12889-023-15204-x)
Supplement: Supplementary file 1 — Supplementary Material 1 [file 12889_2023_15204_MOESM1_ESM.docx]

Burden of *Brucella* spp. in dairy; Rwanda, 2010

Total population

## Incidence

| Food | 2.5% | Mean | 97.5% |
| --- | --- | --- | --- |
| DAIRY | 0.5170000 | 652.0 | 4480 |
| Milk from cattle | 0.4210000 | 597.0 | 4280 |
| Milk from other animals | 0.0001670 | 54.9 | 250 |
| Consumed raw | 0.1800000 | 381.0 | 2870 |
| Fermented by traditional processes (e.g. ikivugoto) | 0.0003060 | 127.0 | 868 |
| Heat treated | 0.0001380 | 31.2 | 176 |
| Fermented by industrial processes | 0.0000222 | 36.4 | 234 |
| Other dairy products | 0.0000001 | 22.1 | 133 |

## Mortality

| Food | 2.5% | Mean | 97.5% |
| --- | --- | --- | --- |
| DAIRY | 0.0024100 | 3.280 | 23.300 |
| Milk from cattle | 0.0020700 | 3.000 | 21.500 |
| Milk from other animals | 0.0000008 | 0.273 | 1.280 |
| Consumed raw | 0.0009010 | 1.920 | 14.400 |
| Fermented by traditional processes (e.g. ikivugoto) | 0.0000016 | 0.634 | 4.500 |
| Heat treated | 0.0000006 | 0.152 | 0.882 |
| Fermented by industrial processes | 0.0000001 | 0.182 | 1.130 |
| Other dairy products | 0.0000000 | 0.109 | 0.646 |

##

## DALY

| Food | 2.5% | Mean | 97.5% |
| --- | --- | --- | --- |
| DAIRY | 0.1560000 | 208.00 | 1460.0 |
| Milk from cattle | 0.1290000 | 191.00 | 1370.0 |
| Milk from other animals | 0.0000512 | 17.40 | 80.8 |
| Consumed raw | 0.0577000 | 122.00 | 912.0 |
| Fermented by traditional processes (e.g. ikivugoto) | 0.0001040 | 40.40 | 285.0 |
| Heat treated | 0.0000406 | 9.69 | 56.1 |
| Fermented by industrial processes | 0.0000072 | 11.60 | 72.4 |
| Other dairy products | 0.0000000 | 6.99 | 41.0 |

## YLL

| Food | 2.5% | Mean | 97.5% |
| --- | --- | --- | --- |
| DAIRY | 0.1370000 | 186.00 | 1320.0 |
| Milk from cattle | 0.1170000 | 170.00 | 1220.0 |
| Milk from other animals | 0.0000457 | 15.50 | 72.8 |
| Consumed raw | 0.0511000 | 109.00 | 816.0 |
| Fermented by traditional processes (e.g. ikivugoto) | 0.0000927 | 35.90 | 255.0 |
| Heat treated | 0.0000364 | 8.64 | 50.0 |
| Fermented by industrial processes | 0.0000062 | 10.30 | 63.9 |
| Other dairy products | 0.0000000 | 6.20 | 36.6 |

## YLD

| Food | 2.5% | Mean | 97.5% |
| --- | --- | --- | --- |
| DAIRY | 0.0161000 | 22.500 | 149.00 |
| Milk from cattle | 0.0127000 | 20.600 | 141.00 |
| Milk from other animals | 0.0000057 | 1.900 | 8.52 |
| Consumed raw | 0.0058700 | 13.100 | 93.40 |
| Fermented by traditional processes (e.g. ikivugoto) | 0.0000109 | 4.440 | 28.60 |
| Heat treated | 0.0000043 | 1.050 | 6.03 |
| Fermented by industrial processes | 0.0000008 | 1.270 | 7.45 |
| Other dairy products | 0.0000000 | 0.789 | 4.48 |

##

## Incidence rate (per 100,000 population)

| Food | 2.5% | Mean | 97.5% |
| --- | --- | --- | --- |
| DAIRY | 0.0047700 | 6.020 | 41.30 |
| Milk from cattle | 0.0038900 | 5.510 | 39.50 |
| Milk from other animals | 0.0000015 | 0.507 | 2.30 |
| Consumed raw | 0.0016600 | 3.520 | 26.50 |
| Fermented by traditional processes (e.g. ikivugoto) | 0.0000028 | 1.170 | 8.01 |
| Heat treated | 0.0000013 | 0.288 | 1.63 |
| Fermented by industrial processes | 0.0000002 | 0.336 | 2.16 |
| Other dairy products | 0.0000000 | 0.204 | 1.23 |

## Mortality rate (per 100,000 population)

| Food | 2.5% | Mean | 97.5% |
| --- | --- | --- | --- |
| DAIRY | 0.0000222 | 0.03020 | 0.21500 |
| Milk from cattle | 0.0000191 | 0.02770 | 0.19900 |
| Milk from other animals | 0.0000000 | 0.00252 | 0.01180 |
| Consumed raw | 0.0000083 | 0.01780 | 0.13300 |
| Fermented by traditional processes (e.g. ikivugoto) | 0.0000000 | 0.00585 | 0.04160 |
| Heat treated | 0.0000000 | 0.00141 | 0.00814 |
| Fermented by industrial processes | 0.0000000 | 0.00168 | 0.01040 |
| Other dairy products | 0.0000000 | 0.00101 | 0.00596 |

##

## DALY rate (per 100,000 population)

| Food | 2.5% | Mean | 97.5% |
| --- | --- | --- | --- |
| DAIRY | 0.0014400 | 1.9200 | 13.500 |
| Milk from cattle | 0.0011900 | 1.7600 | 12.700 |
| Milk from other animals | 0.0000005 | 0.1600 | 0.746 |
| Consumed raw | 0.0005320 | 1.1300 | 8.410 |
| Fermented by traditional processes (e.g. ikivugoto) | 0.0000010 | 0.3730 | 2.630 |
| Heat treated | 0.0000004 | 0.0894 | 0.518 |
| Fermented by industrial processes | 0.0000001 | 0.1070 | 0.668 |
| Other dairy products | 0.0000000 | 0.0645 | 0.378 |

## YLL rate (per 100,000 population)

| Food | 2.5% | Mean | 97.5% |
| --- | --- | --- | --- |
| DAIRY | 0.0012600 | 1.7100 | 12.200 |
| Milk from cattle | 0.0010800 | 1.5700 | 11.300 |
| Milk from other animals | 0.0000004 | 0.1430 | 0.672 |
| Consumed raw | 0.0004710 | 1.0100 | 7.530 |
| Fermented by traditional processes (e.g. ikivugoto) | 0.0000009 | 0.3320 | 2.360 |
| Heat treated | 0.0000003 | 0.0797 | 0.462 |
| Fermented by industrial processes | 0.0000001 | 0.0955 | 0.589 |
| Other dairy products | 0.0000000 | 0.0572 | 0.338 |

## YLD rate (per 100,000 population)

| Food | 2.5% | Mean | 97.5% |
| --- | --- | --- | --- |
| DAIRY | 0.0001480 | 0.20800 | 1.3700 |
| Milk from cattle | 0.0001170 | 0.19000 | 1.3000 |
| Milk from other animals | 0.0000001 | 0.01750 | 0.0786 |
| Consumed raw | 0.0000542 | 0.12100 | 0.8620 |
| Fermented by traditional processes (e.g. ikivugoto) | 0.0000001 | 0.04100 | 0.2640 |
| Heat treated | 0.0000000 | 0.00966 | 0.0557 |
| Fermented by industrial processes | 0.0000000 | 0.01170 | 0.0687 |
| Other dairy products | 0.0000000 | 0.00728 | 0.0413 |

Children under 5 years of age

## Incidence

| Food | 2.5% | Mean | 97.5% |
| --- | --- | --- | --- |
| DAIRY | 0.0057200 | 7.220 | 49.50 |
| Milk from cattle | 0.0046600 | 6.610 | 47.40 |
| Milk from other animals | 0.0000018 | 0.607 | 2.76 |
| Consumed raw | 0.0019900 | 4.220 | 31.70 |
| Fermented by traditional processes (e.g. ikivugoto) | 0.0000034 | 1.400 | 9.61 |
| Heat treated | 0.0000015 | 0.345 | 1.95 |
| Fermented by industrial processes | 0.0000002 | 0.403 | 2.59 |
| Other dairy products | 0.0000000 | 0.244 | 1.47 |

## Mortality

| Food | 2.5% | Mean | 97.5% |
| --- | --- | --- | --- |
| DAIRY | 0.0000266 | 0.03620 | 0.25800 |
| Milk from cattle | 0.0000228 | 0.03320 | 0.23800 |
| Milk from other animals | 0.0000000 | 0.00302 | 0.01420 |
| Consumed raw | 0.0000100 | 0.02130 | 0.15900 |
| Fermented by traditional processes (e.g. ikivugoto) | 0.0000000 | 0.00701 | 0.04980 |
| Heat treated | 0.0000000 | 0.00169 | 0.00976 |
| Fermented by industrial processes | 0.0000000 | 0.00202 | 0.01250 |
| Other dairy products | 0.0000000 | 0.00121 | 0.00715 |

## DALY

| Food | 2.5% | Mean | 97.5% |
| --- | --- | --- | --- |
| DAIRY | 0.0025900 | 3.500 | 24.700 |
| Milk from cattle | 0.0021800 | 3.200 | 23.000 |
| Milk from other animals | 0.0000009 | 0.291 | 1.360 |
| Consumed raw | 0.0009680 | 2.050 | 15.200 |
| Fermented by traditional processes (e.g. ikivugoto) | 0.0000018 | 0.677 | 4.790 |
| Heat treated | 0.0000007 | 0.163 | 0.940 |
| Fermented by industrial processes | 0.0000001 | 0.195 | 1.210 |
| Other dairy products | 0.0000000 | 0.117 | 0.689 |

## YLL

| Food | 2.5% | Mean | 97.5% |
| --- | --- | --- | --- |
| DAIRY | 0.0023900 | 3.250 | 23.100 |
| Milk from cattle | 0.0020500 | 2.980 | 21.300 |
| Milk from other animals | 0.0000008 | 0.270 | 1.270 |
| Consumed raw | 0.0008930 | 1.910 | 14.300 |
| Fermented by traditional processes (e.g. ikivugoto) | 0.0000016 | 0.628 | 4.460 |
| Heat treated | 0.0000006 | 0.151 | 0.874 |
| Fermented by industrial processes | 0.0000001 | 0.181 | 1.120 |
| Other dairy products | 0.0000000 | 0.108 | 0.641 |

## YLD

| Food | 2.5% | Mean | 97.5% |
| --- | --- | --- | --- |
| DAIRY | 0.0001780 | 0.24900 | 1.6400 |
| Milk from cattle | 0.0001400 | 0.22800 | 1.5600 |
| Milk from other animals | 0.0000001 | 0.02100 | 0.0942 |
| Consumed raw | 0.0000650 | 0.14500 | 1.0300 |
| Fermented by traditional processes (e.g. ikivugoto) | 0.0000001 | 0.04910 | 0.3160 |
| Heat treated | 0.0000000 | 0.01160 | 0.0667 |
| Fermented by industrial processes | 0.0000000 | 0.01410 | 0.0824 |
| Other dairy products | 0.0000000 | 0.00872 | 0.0495 |

## Incidence rate (per 100,000 population)

| Food | 2.5% | Mean | 97.5% |
| --- | --- | --- | --- |
| DAIRY | 0.0003200 | 0.4040 | 2.7700 |
| Milk from cattle | 0.0002610 | 0.3700 | 2.6500 |
| Milk from other animals | 0.0000001 | 0.0340 | 0.1550 |
| Consumed raw | 0.0001110 | 0.2360 | 1.7800 |
| Fermented by traditional processes (e.g. ikivugoto) | 0.0000002 | 0.0784 | 0.5380 |
| Heat treated | 0.0000001 | 0.0193 | 0.1090 |
| Fermented by industrial processes | 0.0000000 | 0.0226 | 0.1450 |
| Other dairy products | 0.0000000 | 0.0137 | 0.0824 |

## Mortality rate (per 100,000 population)

| Food | 2.5% | Mean | 97.5% |
| --- | --- | --- | --- |
| DAIRY | 0.0000015 | 0.0020300 | 0.014500 |
| Milk from cattle | 0.0000013 | 0.0018600 | 0.013300 |
| Milk from other animals | 0.0000000 | 0.0001690 | 0.000795 |
| Consumed raw | 0.0000006 | 0.0011900 | 0.008920 |
| Fermented by traditional processes (e.g. ikivugoto) | 0.0000000 | 0.0003930 | 0.002790 |
| Heat treated | 0.0000000 | 0.0000944 | 0.000546 |
| Fermented by industrial processes | 0.0000000 | 0.0001130 | 0.000698 |
| Other dairy products | 0.0000000 | 0.0000677 | 0.000400 |

## DALY rate (per 100,000 population)

| Food | 2.5% | Mean | 97.5% |
| --- | --- | --- | --- |
| DAIRY | 0.0001450 | 0.19600 | 1.3800 |
| Milk from cattle | 0.0001220 | 0.17900 | 1.2900 |
| Milk from other animals | 0.0000000 | 0.01630 | 0.0759 |
| Consumed raw | 0.0000542 | 0.11500 | 0.8510 |
| Fermented by traditional processes (e.g. ikivugoto) | 0.0000001 | 0.03790 | 0.2680 |
| Heat treated | 0.0000000 | 0.00910 | 0.0526 |
| Fermented by industrial processes | 0.0000000 | 0.01090 | 0.0676 |
| Other dairy products | 0.0000000 | 0.00656 | 0.0386 |

## YLL rate (per 100,000 population)

| Food | 2.5% | Mean | 97.5% |
| --- | --- | --- | --- |
| DAIRY | 0.0001340 | 0.18200 | 1.3000 |
| Milk from cattle | 0.0001150 | 0.16700 | 1.1900 |
| Milk from other animals | 0.0000000 | 0.01510 | 0.0712 |
| Consumed raw | 0.0000500 | 0.10700 | 0.7990 |
| Fermented by traditional processes (e.g. ikivugoto) | 0.0000001 | 0.03520 | 0.2500 |
| Heat treated | 0.0000000 | 0.00846 | 0.0490 |
| Fermented by industrial processes | 0.0000000 | 0.01010 | 0.0625 |
| Other dairy products | 0.0000000 | 0.00607 | 0.0359 |

## YLD rate (per 100,000 population)

| Food | 2.5% | Mean | 97.5% |
| --- | --- | --- | --- |
| DAIRY | 0.0000100 | 0.014000 | 0.09210 |
| Milk from cattle | 0.0000079 | 0.012800 | 0.08740 |
| Milk from other animals | 0.0000000 | 0.001180 | 0.00528 |
| Consumed raw | 0.0000036 | 0.008110 | 0.05790 |
| Fermented by traditional processes (e.g. ikivugoto) | 0.0000000 | 0.002750 | 0.01770 |
| Heat treated | 0.0000000 | 0.000648 | 0.00374 |
| Fermented by industrial processes | 0.0000000 | 0.000788 | 0.00461 |
| Other dairy products | 0.0000000 | 0.000489 | 0.00277 |

```

Children over the age of 5 and adults

## Incidence

| Food | 2.5% | Mean | 97.5% |
| --- | --- | --- | --- |
| DAIRY | 0.5110000 | 645.0 | 4430 |
| Milk from cattle | 0.4170000 | 591.0 | 4240 |
| Milk from other animals | 0.0001650 | 54.3 | 247 |
| Consumed raw | 0.1780000 | 377.0 | 2840 |
| Fermented by traditional processes (e.g. ikivugoto) | 0.0003030 | 125.0 | 859 |
| Heat treated | 0.0001370 | 30.8 | 175 |
| Fermented by industrial processes | 0.0000220 | 36.0 | 231 |
| Other dairy products | 0.0000001 | 21.8 | 132 |

## Mortality

| Food | 2.5% | Mean | 97.5% |
| --- | --- | --- | --- |
| DAIRY | 0.0023800 | 3.240 | 23.100 |
| Milk from cattle | 0.0020400 | 2.970 | 21.300 |
| Milk from other animals | 0.0000008 | 0.270 | 1.270 |
| Consumed raw | 0.0008910 | 1.900 | 14.200 |
| Fermented by traditional processes (e.g. ikivugoto) | 0.0000016 | 0.627 | 4.450 |
| Heat treated | 0.0000006 | 0.151 | 0.872 |
| Fermented by industrial processes | 0.0000001 | 0.180 | 1.110 |
| Other dairy products | 0.0000000 | 0.108 | 0.639 |

## DALY

| Food | 2.5% | Mean | 97.5% |
| --- | --- | --- | --- |
| DAIRY | 0.1530000 | 205.00 | 1440.0 |
| Milk from cattle | 0.1270000 | 188.00 | 1350.0 |
| Milk from other animals | 0.0000503 | 17.10 | 79.4 |
| Consumed raw | 0.0567000 | 120.00 | 896.0 |
| Fermented by traditional processes (e.g. ikivugoto) | 0.0001020 | 39.70 | 280.0 |
| Heat treated | 0.0000399 | 9.52 | 55.1 |
| Fermented by industrial processes | 0.0000070 | 11.40 | 71.2 |
| Other dairy products | 0.0000000 | 6.87 | 40.3 |

## YLL

| Food | 2.5% | Mean | 97.5% |
| --- | --- | --- | --- |
| DAIRY | 0.1340000 | 182.00 | 1300.0 |
| Milk from cattle | 0.1150000 | 167.00 | 1200.0 |
| Milk from other animals | 0.0000449 | 15.20 | 71.5 |
| Consumed raw | 0.0502000 | 107.00 | 802.0 |
| Fermented by traditional processes (e.g. ikivugoto) | 0.0000910 | 35.30 | 251.0 |
| Heat treated | 0.0000357 | 8.49 | 49.1 |
| Fermented by industrial processes | 0.0000061 | 10.20 | 62.8 |
| Other dairy products | 0.0000000 | 6.09 | 36.0 |

## YLD

| Food | 2.5% | Mean | 97.5% |
| --- | --- | --- | --- |
| DAIRY | 0.0159000 | 22.30 | 147.00 |
| Milk from cattle | 0.0125000 | 20.40 | 140.00 |
| Milk from other animals | 0.0000056 | 1.88 | 8.43 |
| Consumed raw | 0.0058100 | 12.90 | 92.40 |
| Fermented by traditional processes (e.g. ikivugoto) | 0.0000108 | 4.39 | 28.30 |
| Heat treated | 0.0000043 | 1.04 | 5.97 |
| Fermented by industrial processes | 0.0000008 | 1.26 | 7.37 |
| Other dairy products | 0.0000000 | 0.78 | 4.43 |

## Incidence rate (per 100,000 population)

| Food | 2.5% | Mean | 97.5% |
| --- | --- | --- | --- |
| DAIRY | 0.0056500 | 7.130 | 48.90 |
| Milk from cattle | 0.0046000 | 6.530 | 46.80 |
| Milk from other animals | 0.0000018 | 0.600 | 2.73 |
| Consumed raw | 0.0019600 | 4.160 | 31.40 |
| Fermented by traditional processes (e.g. ikivugoto) | 0.0000033 | 1.380 | 9.49 |
| Heat treated | 0.0000015 | 0.341 | 1.93 |
| Fermented by industrial processes | 0.0000002 | 0.398 | 2.55 |
| Other dairy products | 0.0000000 | 0.241 | 1.45 |

## Mortality rate (per 100,000 population)

| Food | 2.5% | Mean | 97.5% |
| --- | --- | --- | --- |
| DAIRY | 0.0000263 | 0.03580 | 0.25500 |
| Milk from cattle | 0.0000226 | 0.03280 | 0.23500 |
| Milk from other animals | 0.0000000 | 0.00298 | 0.01400 |
| Consumed raw | 0.0000098 | 0.02100 | 0.15700 |
| Fermented by traditional processes (e.g. ikivugoto) | 0.0000000 | 0.00693 | 0.04920 |
| Heat treated | 0.0000000 | 0.00166 | 0.00964 |
| Fermented by industrial processes | 0.0000000 | 0.00199 | 0.01230 |
| Other dairy products | 0.0000000 | 0.00119 | 0.00706 |

## DALY rate (per 100,000 population)

| Food | 2.5% | Mean | 97.5% |
| --- | --- | --- | --- |
| DAIRY | 0.0017000 | 2.2600 | 15.900 |
| Milk from cattle | 0.0014000 | 2.0700 | 14.900 |
| Milk from other animals | 0.0000006 | 0.1890 | 0.878 |
| Consumed raw | 0.0006270 | 1.3300 | 9.900 |
| Fermented by traditional processes (e.g. ikivugoto) | 0.0000011 | 0.4390 | 3.090 |
| Heat treated | 0.0000004 | 0.1050 | 0.609 |
| Fermented by industrial processes | 0.0000001 | 0.1260 | 0.786 |
| Other dairy products | 0.0000000 | 0.0759 | 0.445 |

## YLL rate (per 100,000 population)

| Food | 2.5% | Mean | 97.5% |
| --- | --- | --- | --- |
| DAIRY | 0.0014800 | 2.0200 | 14.400 |
| Milk from cattle | 0.0012700 | 1.8500 | 13.200 |
| Milk from other animals | 0.0000005 | 0.1680 | 0.790 |
| Consumed raw | 0.0005540 | 1.1800 | 8.860 |
| Fermented by traditional processes (e.g. ikivugoto) | 0.0000010 | 0.3900 | 2.770 |
| Heat treated | 0.0000004 | 0.0938 | 0.543 |
| Fermented by industrial processes | 0.0000001 | 0.1120 | 0.693 |
| Other dairy products | 0.0000000 | 0.0673 | 0.398 |

## YLD rate (per 100,000 population)

| Food | 2.5% | Mean | 97.5% |
| --- | --- | --- | --- |
| DAIRY | 0.0001760 | 0.24600 | 1.6200 |
| Milk from cattle | 0.0001390 | 0.22500 | 1.5400 |
| Milk from other animals | 0.0000001 | 0.02070 | 0.0931 |
| Consumed raw | 0.0000642 | 0.14300 | 1.0200 |
| Fermented by traditional processes (e.g. ikivugoto) | 0.0000001 | 0.04850 | 0.3130 |
| Heat treated | 0.0000000 | 0.01140 | 0.0659 |
| Fermented by industrial processes | 0.0000000 | 0.01390 | 0.0814 |
| Other dairy products | 0.0000000 | 0.00862 | 0.0489 |

```
