# Supplementary material for: Estimates of disease burden caused by foodborne pathogens in contaminated dairy products in Rwanda
Source: BMC Public Health. 2023 Apr 6;23:657. doi: 10.1186/s12889-023-15204-x (PMC10077627; doi:10.1186/s12889-023-15204-x)
Supplement: Supplementary file 2 — Supplementary Material 2 [file 12889_2023_15204_MOESM2_ESM.docx]

Burden of *Campylobacter* spp. in dairy; Rwanda, 2010

Total population

## Incidence

| Food | 2.5% | Mean | 97.5% |
| --- | --- | --- | --- |
| DAIRY | 898.00000 | 44600 | 172000 |
| Milk from cattle | 188.00000 | 20800 | 81700 |
| Milk from other animals | 0.00478 | 2940 | 21600 |
| Consumed raw | 0.00635 | 7330 | 33500 |
| Fermented by traditional processes (e.g. ikivugoto) | 0.01090 | 4820 | 23000 |
| Heat treated | 0.44700 | 1650 | 13800 |
| Fermented by industrial processes | 4.85000 | 5190 | 25900 |
| Other dairy products | 0.56000 | 1820 | 11600 |

## Mortality

| Food | 2.5% | Mean | 97.5% |
| --- | --- | --- | --- |
| DAIRY | 0.4440000 | 12.500 | 31.80 |
| Milk from cattle | 0.1140000 | 5.820 | 14.90 |
| Milk from other animals | 0.0000029 | 0.823 | 4.94 |
| Consumed raw | 0.0000034 | 2.040 | 6.84 |
| Fermented by traditional processes (e.g. ikivugoto) | 0.0000047 | 1.370 | 5.11 |
| Heat treated | 0.0002450 | 0.456 | 3.47 |
| Fermented by industrial processes | 0.0026200 | 1.440 | 5.46 |
| Other dairy products | 0.0003150 | 0.513 | 3.07 |

##

## DALY

| Food | 2.5% | Mean | 97.5% |
| --- | --- | --- | --- |
| DAIRY | 41.900000 | 1150.0 | 2910 |
| Milk from cattle | 10.100000 | 536.0 | 1370 |
| Milk from other animals | 0.000266 | 75.8 | 455 |
| Consumed raw | 0.000311 | 188.0 | 630 |
| Fermented by traditional processes (e.g. ikivugoto) | 0.000439 | 126.0 | 468 |
| Heat treated | 0.022800 | 42.0 | 317 |
| Fermented by industrial processes | 0.232000 | 133.0 | 504 |
| Other dairy products | 0.028900 | 47.3 | 283 |

## YLL

| Food | 2.5% | Mean | 97.5% |
| --- | --- | --- | --- |
| DAIRY | 36.500000 | 1020.0 | 2620 |
| Milk from cattle | 9.360000 | 476.0 | 1230 |
| Milk from other animals | 0.000236 | 67.3 | 408 |
| Consumed raw | 0.000274 | 167.0 | 563 |
| Fermented by traditional processes (e.g. ikivugoto) | 0.000385 | 112.0 | 421 |
| Heat treated | 0.020300 | 37.3 | 283 |
| Fermented by industrial processes | 0.213000 | 118.0 | 451 |
| Other dairy products | 0.025100 | 42.0 | 251 |

## YLD

| Food | 2.5% | Mean | 97.5% |
| --- | --- | --- | --- |
| DAIRY | 4.8100000 | 129.00 | 373.0 |
| Milk from cattle | 1.0100000 | 60.00 | 177.0 |
| Milk from other animals | 0.0000266 | 8.47 | 54.4 |
| Consumed raw | 0.0000336 | 21.10 | 78.3 |
| Fermented by traditional processes (e.g. ikivugoto) | 0.0000436 | 14.00 | 54.8 |
| Heat treated | 0.0024700 | 4.76 | 36.1 |
| Fermented by industrial processes | 0.0208000 | 14.90 | 61.2 |
| Other dairy products | 0.0030300 | 5.27 | 31.4 |

##

## Incidence rate (per 100,000 population)

| Food | 2.5% | Mean | 97.5% |
| --- | --- | --- | --- |
| DAIRY | 8.2800000 | 411.0 | 1590 |
| Milk from cattle | 1.7400000 | 192.0 | 754 |
| Milk from other animals | 0.0000441 | 27.1 | 200 |
| Consumed raw | 0.0000586 | 67.6 | 309 |
| Fermented by traditional processes (e.g. ikivugoto) | 0.0001010 | 44.5 | 212 |
| Heat treated | 0.0041300 | 15.3 | 128 |
| Fermented by industrial processes | 0.0447000 | 47.9 | 239 |
| Other dairy products | 0.0051700 | 16.8 | 107 |

## Mortality rate (per 100,000 population)

| Food | 2.5% | Mean | 97.5% |
| --- | --- | --- | --- |
| DAIRY | 0.0040900 | 0.11500 | 0.2940 |
| Milk from cattle | 0.0010500 | 0.05370 | 0.1380 |
| Milk from other animals | 0.0000000 | 0.00759 | 0.0456 |
| Consumed raw | 0.0000000 | 0.01890 | 0.0631 |
| Fermented by traditional processes (e.g. ikivugoto) | 0.0000000 | 0.01260 | 0.0471 |
| Heat treated | 0.0000023 | 0.00420 | 0.0320 |
| Fermented by industrial processes | 0.0000242 | 0.01330 | 0.0504 |
| Other dairy products | 0.0000029 | 0.00474 | 0.0283 |

##

## DALY rate (per 100,000 population)

| Food | 2.5% | Mean | 97.5% |
| --- | --- | --- | --- |
| DAIRY | 0.3870000 | 10.600 | 26.90 |
| Milk from cattle | 0.0933000 | 4.950 | 12.60 |
| Milk from other animals | 0.0000024 | 0.699 | 4.20 |
| Consumed raw | 0.0000029 | 1.740 | 5.81 |
| Fermented by traditional processes (e.g. ikivugoto) | 0.0000040 | 1.160 | 4.32 |
| Heat treated | 0.0002100 | 0.388 | 2.93 |
| Fermented by industrial processes | 0.0021400 | 1.220 | 4.65 |
| Other dairy products | 0.0002670 | 0.436 | 2.61 |

## YLL rate (per 100,000 population)

| Food | 2.5% | Mean | 97.5% |
| --- | --- | --- | --- |
| DAIRY | 0.3370000 | 9.410 | 24.20 |
| Milk from cattle | 0.0863000 | 4.390 | 11.40 |
| Milk from other animals | 0.0000022 | 0.621 | 3.77 |
| Consumed raw | 0.0000025 | 1.540 | 5.20 |
| Fermented by traditional processes (e.g. ikivugoto) | 0.0000036 | 1.030 | 3.88 |
| Heat treated | 0.0001870 | 0.344 | 2.61 |
| Fermented by industrial processes | 0.0019600 | 1.090 | 4.16 |
| Other dairy products | 0.0002320 | 0.388 | 2.31 |

## YLD rate (per 100,000 population)

| Food | 2.5% | Mean | 97.5% |
| --- | --- | --- | --- |
| DAIRY | 0.0444000 | 1.1900 | 3.440 |
| Milk from cattle | 0.0092900 | 0.5540 | 1.640 |
| Milk from other animals | 0.0000002 | 0.0781 | 0.502 |
| Consumed raw | 0.0000003 | 0.1950 | 0.722 |
| Fermented by traditional processes (e.g. ikivugoto) | 0.0000004 | 0.1290 | 0.506 |
| Heat treated | 0.0000228 | 0.0440 | 0.333 |
| Fermented by industrial processes | 0.0001920 | 0.1380 | 0.565 |
| Other dairy products | 0.0000279 | 0.0486 | 0.289 |

```

Children under the age of 5

## Incidence

| Food | 2.5% | Mean | 97.5% |
| --- | --- | --- | --- |
| DAIRY | 61.40000 | 34700 | 154000 |
| Milk from cattle | 33.10000 | 31000 | 141000 |
| Milk from other animals | 0.00265 | 3730 | 27500 |
| Consumed raw | 0.00579 | 10900 | 57900 |
| Fermented by traditional processes (e.g. ikivugoto) | 0.01090 | 7290 | 40700 |
| Heat treated | 0.12300 | 2430 | 20900 |
| Fermented by industrial processes | 2.12000 | 7520 | 41900 |
| Other dairy products | 0.30300 | 2770 | 19800 |

## Mortality

| Food | 2.5% | Mean | 97.5% |
| --- | --- | --- | --- |
| DAIRY | 0.2980000 | 8.380 | 22.40 |
| Milk from cattle | 0.1640000 | 7.490 | 20.70 |
| Milk from other animals | 0.0000038 | 0.898 | 5.23 |
| Consumed raw | 0.0000037 | 2.660 | 9.36 |
| Fermented by traditional processes (e.g. ikivugoto) | 0.0000050 | 1.760 | 6.83 |
| Heat treated | 0.0004410 | 0.579 | 4.39 |
| Fermented by industrial processes | 0.0037100 | 1.830 | 7.36 |
| Other dairy products | 0.0010100 | 0.655 | 4.00 |

## DALY

| Food | 2.5% | Mean | 97.5% |
| --- | --- | --- | --- |
| DAIRY | 29.500000 | 816.0 | 2170 |
| Milk from cattle | 15.400000 | 729.0 | 1980 |
| Milk from other animals | 0.000358 | 87.4 | 513 |
| Consumed raw | 0.000364 | 259.0 | 905 |
| Fermented by traditional processes (e.g. ikivugoto) | 0.000505 | 171.0 | 671 |
| Heat treated | 0.041000 | 56.4 | 429 |
| Fermented by industrial processes | 0.362000 | 178.0 | 703 |
| Other dairy products | 0.099900 | 63.9 | 389 |

## YLL

| Food | 2.5% | Mean | 97.5% |
| --- | --- | --- | --- |
| DAIRY | 26.700000 | 751.0 | 2010 |
| Milk from cattle | 14.700000 | 671.0 | 1860 |
| Milk from other animals | 0.000341 | 80.4 | 468 |
| Consumed raw | 0.000329 | 239.0 | 839 |
| Fermented by traditional processes (e.g. ikivugoto) | 0.000449 | 158.0 | 612 |
| Heat treated | 0.039500 | 51.9 | 393 |
| Fermented by industrial processes | 0.332000 | 164.0 | 659 |
| Other dairy products | 0.090500 | 58.7 | 358 |

## YLD

| Food | 2.5% | Mean | 97.5% |
| --- | --- | --- | --- |
| DAIRY | 1.5300000 | 65.10 | 256.0 |
| Milk from cattle | 0.7360000 | 58.10 | 237.0 |
| Milk from other animals | 0.0000143 | 7.01 | 48.7 |
| Consumed raw | 0.0000182 | 20.60 | 97.8 |
| Fermented by traditional processes (e.g. ikivugoto) | 0.0000282 | 13.70 | 68.5 |
| Heat treated | 0.0015100 | 4.56 | 37.3 |
| Fermented by industrial processes | 0.0161000 | 14.10 | 72.5 |
| Other dairy products | 0.0045800 | 5.20 | 34.6 |

## Incidence rate (per 100,000 population)

| Food | 2.5% | Mean | 97.5% |
| --- | --- | --- | --- |
| DAIRY | 3.440000 | 1940 | 8610 |
| Milk from cattle | 1.850000 | 1730 | 7900 |
| Milk from other animals | 0.000149 | 209 | 1540 |
| Consumed raw | 0.000324 | 613 | 3240 |
| Fermented by traditional processes (e.g. ikivugoto) | 0.000609 | 408 | 2280 |
| Heat treated | 0.006880 | 136 | 1170 |
| Fermented by industrial processes | 0.119000 | 421 | 2350 |
| Other dairy products | 0.017000 | 155 | 1110 |

## Mortality rate (per 100,000 population)

| Food | 2.5% | Mean | 97.5% |
| --- | --- | --- | --- |
| DAIRY | 0.0167000 | 0.4690 | 1.260 |
| Milk from cattle | 0.0091900 | 0.4190 | 1.160 |
| Milk from other animals | 0.0000002 | 0.0503 | 0.293 |
| Consumed raw | 0.0000002 | 0.1490 | 0.524 |
| Fermented by traditional processes (e.g. ikivugoto) | 0.0000003 | 0.0985 | 0.383 |
| Heat treated | 0.0000247 | 0.0324 | 0.246 |
| Fermented by industrial processes | 0.0002080 | 0.1020 | 0.412 |
| Other dairy products | 0.0000566 | 0.0367 | 0.224 |

## DALY rate (per 100,000 population)

| Food | 2.5% | Mean | 97.5% |
| --- | --- | --- | --- |
| DAIRY | 1.6500000 | 45.70 | 122.0 |
| Milk from cattle | 0.8650000 | 40.80 | 111.0 |
| Milk from other animals | 0.0000200 | 4.90 | 28.7 |
| Consumed raw | 0.0000204 | 14.50 | 50.7 |
| Fermented by traditional processes (e.g. ikivugoto) | 0.0000283 | 9.59 | 37.6 |
| Heat treated | 0.0023000 | 3.16 | 24.0 |
| Fermented by industrial processes | 0.0203000 | 9.97 | 39.4 |
| Other dairy products | 0.0056000 | 3.58 | 21.8 |

## YLL rate (per 100,000 population)

| Food | 2.5% | Mean | 97.5% |
| --- | --- | --- | --- |
| DAIRY | 1.5000000 | 42.10 | 112.0 |
| Milk from cattle | 0.8240000 | 37.60 | 104.0 |
| Milk from other animals | 0.0000191 | 4.50 | 26.2 |
| Consumed raw | 0.0000184 | 13.40 | 47.0 |
| Fermented by traditional processes (e.g. ikivugoto) | 0.0000251 | 8.82 | 34.3 |
| Heat treated | 0.0022100 | 2.90 | 22.0 |
| Fermented by industrial processes | 0.0186000 | 9.18 | 36.9 |
| Other dairy products | 0.0050700 | 3.29 | 20.0 |

## YLD rate (per 100,000 population)

| Food | 2.5% | Mean | 97.5% |
| --- | --- | --- | --- |
| DAIRY | 0.0858000 | 3.650 | 14.30 |
| Milk from cattle | 0.0412000 | 3.250 | 13.30 |
| Milk from other animals | 0.0000008 | 0.392 | 2.73 |
| Consumed raw | 0.0000010 | 1.150 | 5.47 |
| Fermented by traditional processes (e.g. ikivugoto) | 0.0000016 | 0.765 | 3.84 |
| Heat treated | 0.0000847 | 0.255 | 2.09 |
| Fermented by industrial processes | 0.0008990 | 0.792 | 4.06 |
| Other dairy products | 0.0002560 | 0.291 | 1.94 |

```

Children over the age of 5 and adults

## Incidence

|  | Food | 2.5% | Mean | 97.5% |
| --- | --- | --- | --- | --- |
| 17 | DAIRY | 160.0000 | 9900 | 40000 |
| 23 | Milk from cattle | 97.0000 | 8800 | 36000 |
| 24 | Milk from other animals | 0.0021 | 1000 | 7400 |
| 22 | Consumed raw | 0.0028 | 3100 | 15000 |
| 19 | Fermented by traditional processes (e.g. ikivugoto) | 0.0034 | 2100 | 10000 |
| 20 | Heat treated | 0.1200 | 710 | 5800 |
| 18 | Fermented by industrial processes | 1.4000 | 2100 | 11000 |
| 21 | Other dairy products | 0.2600 | 750 | 5100 |

## Mortality

|  | Food | 2.5% | Mean | 97.5% |
| --- | --- | --- | --- | --- |
| 17 | DAIRY | 0.1500000 | 4.10 | 10.0 |
| 23 | Milk from cattle | 0.0780000 | 3.60 | 9.3 |
| 24 | Milk from other animals | 0.0000019 | 0.43 | 2.4 |
| 22 | Consumed raw | 0.0000022 | 1.30 | 4.3 |
| 19 | Fermented by traditional processes (e.g. ikivugoto) | 0.0000024 | 0.87 | 3.2 |
| 20 | Heat treated | 0.0001300 | 0.29 | 2.1 |
| 18 | Fermented by industrial processes | 0.0012000 | 0.89 | 3.3 |
| 21 | Other dairy products | 0.0002700 | 0.32 | 1.9 |

## DALY

|  | Food | 2.5% | Mean | 97.5% |
| --- | --- | --- | --- | --- |
| 17 | DAIRY | 12.00000 | 330 | 810 |
| 23 | Milk from cattle | 6.30000 | 300 | 750 |
| 24 | Milk from other animals | 0.00016 | 35 | 200 |
| 22 | Consumed raw | 0.00018 | 100 | 350 |
| 19 | Fermented by traditional processes (e.g. ikivugoto) | 0.00020 | 71 | 260 |
| 20 | Heat treated | 0.01000 | 23 | 170 |
| 18 | Fermented by industrial processes | 0.09400 | 72 | 260 |
| 21 | Other dairy products | 0.02100 | 26 | 150 |

## YLL

|  | Food | 2.5% | Mean | 97.5% |
| --- | --- | --- | --- | --- |
| 17 | DAIRY | 9.80000 | 270 | 660 |
| 23 | Milk from cattle | 5.10000 | 240 | 610 |
| 24 | Milk from other animals | 0.00013 | 29 | 160 |
| 22 | Consumed raw | 0.00014 | 84 | 280 |
| 19 | Fermented by traditional processes (e.g. ikivugoto) | 0.00015 | 57 | 210 |
| 20 | Heat treated | 0.00820 | 19 | 140 |
| 18 | Fermented by industrial processes | 0.07800 | 58 | 210 |
| 21 | Other dairy products | 0.01800 | 21 | 120 |

## YLD

|  | Food | 2.5% | Mean | 97.5% |
| --- | --- | --- | --- | --- |
| 17 | DAIRY | 2.300000 | 63.0 | 160 |
| 23 | Milk from cattle | 1.200000 | 57.0 | 150 |
| 24 | Milk from other animals | 0.000031 | 6.7 | 38 |
| 22 | Consumed raw | 0.000033 | 20.0 | 65 |
| 19 | Fermented by traditional processes (e.g. ikivugoto) | 0.000038 | 13.0 | 49 |
| 20 | Heat treated | 0.002100 | 4.5 | 33 |
| 18 | Fermented by industrial processes | 0.017000 | 14.0 | 51 |
| 21 | Other dairy products | 0.003800 | 5.0 | 29 |

## Incidence rate (per 100,000 population)

|  | Food | 2.5% | Mean | 97.5% |
| --- | --- | --- | --- | --- |
| 17 | DAIRY | 1.800000 | 110.0 | 440 |
| 23 | Milk from cattle | 1.100000 | 98.0 | 400 |
| 24 | Milk from other animals | 0.000023 | 12.0 | 82 |
| 22 | Consumed raw | 0.000031 | 34.0 | 160 |
| 19 | Fermented by traditional processes (e.g. ikivugoto) | 0.000037 | 23.0 | 110 |
| 20 | Heat treated | 0.001400 | 7.8 | 64 |
| 18 | Fermented by industrial processes | 0.016000 | 24.0 | 120 |
| 21 | Other dairy products | 0.002900 | 8.3 | 56 |

## Mortality rate (per 100,000 population)

|  | Food | 2.5% | Mean | 97.5% |
| --- | --- | --- | --- | --- |
| 17 | DAIRY | 0.001600000 | 0.0450 | 0.110 |
| 23 | Milk from cattle | 0.000860000 | 0.0400 | 0.100 |
| 24 | Milk from other animals | 0.000000021 | 0.0048 | 0.027 |
| 22 | Consumed raw | 0.000000024 | 0.0140 | 0.048 |
| 19 | Fermented by traditional processes (e.g. ikivugoto) | 0.000000026 | 0.0096 | 0.036 |
| 20 | Heat treated | 0.000001400 | 0.0032 | 0.023 |
| 18 | Fermented by industrial processes | 0.000013000 | 0.0098 | 0.036 |
| 21 | Other dairy products | 0.000003000 | 0.0035 | 0.021 |

## DALY rate (per 100,000 population)

|  | Food | 2.5% | Mean | 97.5% |
| --- | --- | --- | --- | --- |
| 17 | DAIRY | 0.1400000 | 3.70 | 9.0 |
| 23 | Milk from cattle | 0.0690000 | 3.30 | 8.3 |
| 24 | Milk from other animals | 0.0000018 | 0.39 | 2.2 |
| 22 | Consumed raw | 0.0000020 | 1.20 | 3.8 |
| 19 | Fermented by traditional processes (e.g. ikivugoto) | 0.0000022 | 0.78 | 2.9 |
| 20 | Heat treated | 0.0001100 | 0.26 | 1.9 |
| 18 | Fermented by industrial processes | 0.0010000 | 0.80 | 2.9 |
| 21 | Other dairy products | 0.0002400 | 0.29 | 1.7 |

## YLL rate (per 100,000 population)

|  | Food | 2.5% | Mean | 97.5% |
| --- | --- | --- | --- | --- |
| 17 | DAIRY | 0.1100000 | 3.00 | 7.3 |
| 23 | Milk from cattle | 0.0560000 | 2.60 | 6.8 |
| 24 | Milk from other animals | 0.0000014 | 0.32 | 1.8 |
| 22 | Consumed raw | 0.0000016 | 0.93 | 3.1 |
| 19 | Fermented by traditional processes (e.g. ikivugoto) | 0.0000017 | 0.63 | 2.4 |
| 20 | Heat treated | 0.0000910 | 0.21 | 1.5 |
| 18 | Fermented by industrial processes | 0.0008600 | 0.65 | 2.4 |
| 21 | Other dairy products | 0.0002000 | 0.23 | 1.4 |

## YLD rate (per 100,000 population)

|  | Food | 2.5% | Mean | 97.5% |
| --- | --- | --- | --- | --- |
| 17 | DAIRY | 0.02600000 | 0.700 | 1.70 |
| 23 | Milk from cattle | 0.01400000 | 0.630 | 1.60 |
| 24 | Milk from other animals | 0.00000034 | 0.075 | 0.42 |
| 22 | Consumed raw | 0.00000037 | 0.220 | 0.72 |
| 19 | Fermented by traditional processes (e.g. ikivugoto) | 0.00000042 | 0.150 | 0.54 |
| 20 | Heat treated | 0.00002300 | 0.049 | 0.37 |
| 18 | Fermented by industrial processes | 0.00018000 | 0.150 | 0.57 |
| 21 | Other dairy products | 0.00004200 | 0.055 | 0.32 |

```
