# Supplementary material for: Estimates of disease burden caused by foodborne pathogens in contaminated dairy products in Rwanda
Source: BMC Public Health. 2023 Apr 6;23:657. doi: 10.1186/s12889-023-15204-x (PMC10077627; doi:10.1186/s12889-023-15204-x)
Supplement: Supplementary file 3 — Supplementary Material 3 [file 12889_2023_15204_MOESM3_ESM.docx]

Burden of *Cryptosporidium* spp. in dairy; Rwanda, 2010

Total population

## Incidence

| Food | 2.5% | Mean | 97.5% |
| --- | --- | --- | --- |
| DAIRY | 0 | 4470 | 29800 |
| Milk from cattle | 0 | 3980 | 26200 |
| Milk from other animals | 0 | 491 | 4570 |
| Consumed raw | 0 | 1920 | 13400 |
| Fermented by traditional processes (e.g. ikivugoto) | 0 | 1050 | 7690 |
| Heat treated | 0 | 232 | 2310 |
| Fermented by industrial processes | 0 | 455 | 3620 |
| Other dairy products | 0 | 323 | 2980 |

## Mortality

| Food | 2.5% | Mean | 97.5% |
| --- | --- | --- | --- |
| DAIRY | 0 | 3.060 | 18.00 |
| Milk from cattle | 0 | 2.730 | 16.40 |
| Milk from other animals | 0 | 0.337 | 3.09 |
| Consumed raw | 0 | 1.320 | 8.50 |
| Fermented by traditional processes (e.g. ikivugoto) | 0 | 0.711 | 4.85 |
| Heat treated | 0 | 0.157 | 1.63 |
| Fermented by industrial processes | 0 | 0.311 | 2.36 |
| Other dairy products | 0 | 0.225 | 1.95 |

##

## DALY

| Food | 2.5% | Mean | 97.5% |
| --- | --- | --- | --- |
| DAIRY | 0 | 254.0 | 1500 |
| Milk from cattle | 0 | 226.0 | 1370 |
| Milk from other animals | 0 | 28.0 | 258 |
| Consumed raw | 0 | 110.0 | 710 |
| Fermented by traditional processes (e.g. ikivugoto) | 0 | 59.0 | 404 |
| Heat treated | 0 | 13.0 | 135 |
| Fermented by industrial processes | 0 | 25.8 | 193 |
| Other dairy products | 0 | 18.7 | 162 |

## YLL

| Food | 2.5% | Mean | 97.5% |
| --- | --- | --- | --- |
| DAIRY | 0 | 250.0 | 1480 |
| Milk from cattle | 0 | 222.0 | 1340 |
| Milk from other animals | 0 | 27.5 | 253 |
| Consumed raw | 0 | 108.0 | 698 |
| Fermented by traditional processes (e.g. ikivugoto) | 0 | 58.0 | 394 |
| Heat treated | 0 | 12.8 | 132 |
| Fermented by industrial processes | 0 | 25.4 | 190 |
| Other dairy products | 0 | 18.4 | 158 |

## YLD

| Food | 2.5% | Mean | 97.5% |
| --- | --- | --- | --- |
| DAIRY | 0 | 4.540 | 29.50 |
| Milk from cattle | 0 | 4.040 | 26.50 |
| Milk from other animals | 0 | 0.501 | 4.58 |
| Consumed raw | 0 | 1.950 | 13.50 |
| Fermented by traditional processes (e.g. ikivugoto) | 0 | 1.060 | 8.00 |
| Heat treated | 0 | 0.236 | 2.33 |
| Fermented by industrial processes | 0 | 0.460 | 3.58 |
| Other dairy products | 0 | 0.328 | 2.99 |

##

## Incidence rate (per 100,000 population)

| Food | 2.5% | Mean | 97.5% |
| --- | --- | --- | --- |
| DAIRY | 0 | 41.20 | 275.0 |
| Milk from cattle | 0 | 36.70 | 242.0 |
| Milk from other animals | 0 | 4.53 | 42.1 |
| Consumed raw | 0 | 17.70 | 124.0 |
| Fermented by traditional processes (e.g. ikivugoto) | 0 | 9.66 | 71.0 |
| Heat treated | 0 | 2.14 | 21.3 |
| Fermented by industrial processes | 0 | 4.20 | 33.4 |
| Other dairy products | 0 | 2.98 | 27.5 |

## Mortality rate (per 100,000 population)

| Food | 2.5% | Mean | 97.5% |
| --- | --- | --- | --- |
| DAIRY | 0 | 0.02830 | 0.1660 |
| Milk from cattle | 0 | 0.02520 | 0.1510 |
| Milk from other animals | 0 | 0.00311 | 0.0285 |
| Consumed raw | 0 | 0.01220 | 0.0784 |
| Fermented by traditional processes (e.g. ikivugoto) | 0 | 0.00656 | 0.0447 |
| Heat treated | 0 | 0.00145 | 0.0150 |
| Fermented by industrial processes | 0 | 0.00287 | 0.0218 |
| Other dairy products | 0 | 0.00208 | 0.0180 |

##

## DALY rate (per 100,000 population)

| Food | 2.5% | Mean | 97.5% |
| --- | --- | --- | --- |
| DAIRY | 0 | 2.350 | 13.90 |
| Milk from cattle | 0 | 2.090 | 12.60 |
| Milk from other animals | 0 | 0.258 | 2.38 |
| Consumed raw | 0 | 1.010 | 6.55 |
| Fermented by traditional processes (e.g. ikivugoto) | 0 | 0.545 | 3.73 |
| Heat treated | 0 | 0.120 | 1.24 |
| Fermented by industrial processes | 0 | 0.238 | 1.78 |
| Other dairy products | 0 | 0.173 | 1.49 |

## YLL rate (per 100,000 population)

| Food | 2.5% | Mean | 97.5% |
| --- | --- | --- | --- |
| DAIRY | 0 | 2.310 | 13.70 |
| Milk from cattle | 0 | 2.050 | 12.40 |
| Milk from other animals | 0 | 0.254 | 2.33 |
| Consumed raw | 0 | 0.995 | 6.44 |
| Fermented by traditional processes (e.g. ikivugoto) | 0 | 0.535 | 3.63 |
| Heat treated | 0 | 0.118 | 1.22 |
| Fermented by industrial processes | 0 | 0.234 | 1.76 |
| Other dairy products | 0 | 0.170 | 1.46 |

## YLD rate (per 100,000 population)

| Food | 2.5% | Mean | 97.5% |
| --- | --- | --- | --- |
| DAIRY | 0 | 0.04190 | 0.2720 |
| Milk from cattle | 0 | 0.03730 | 0.2440 |
| Milk from other animals | 0 | 0.00462 | 0.0423 |
| Consumed raw | 0 | 0.01800 | 0.1250 |
| Fermented by traditional processes (e.g. ikivugoto) | 0 | 0.00979 | 0.0738 |
| Heat treated | 0 | 0.00218 | 0.0215 |
| Fermented by industrial processes | 0 | 0.00425 | 0.0331 |
| Other dairy products | 0 | 0.00303 | 0.0276 |

```

Children under the age of 5 population

## Incidence

| Food | 2.5% | Mean | 97.5% |
| --- | --- | --- | --- |
| DAIRY | 0 | 3660 | 24200 |
| Milk from cattle | 0 | 3260 | 21900 |
| Milk from other animals | 0 | 402 | 3790 |
| Consumed raw | 0 | 1570 | 11300 |
| Fermented by traditional processes (e.g. ikivugoto) | 0 | 858 | 6400 |
| Heat treated | 0 | 191 | 1840 |
| Fermented by industrial processes | 0 | 372 | 2920 |
| Other dairy products | 0 | 267 | 2420 |

## Mortality

| Food | 2.5% | Mean | 97.5% |
| --- | --- | --- | --- |
| DAIRY | 0 | 2.000 | 12.30 |
| Milk from cattle | 0 | 1.780 | 11.30 |
| Milk from other animals | 0 | 0.221 | 2.02 |
| Consumed raw | 0 | 0.862 | 5.80 |
| Fermented by traditional processes (e.g. ikivugoto) | 0 | 0.464 | 3.26 |
| Heat treated | 0 | 0.102 | 1.07 |
| Fermented by industrial processes | 0 | 0.203 | 1.55 |
| Other dairy products | 0 | 0.147 | 1.27 |

## DALY

| Food | 2.5% | Mean | 97.5% |
| --- | --- | --- | --- |
| DAIRY | 0 | 183.00 | 1120.0 |
| Milk from cattle | 0 | 163.00 | 1030.0 |
| Milk from other animals | 0 | 20.20 | 186.0 |
| Consumed raw | 0 | 79.00 | 531.0 |
| Fermented by traditional processes (e.g. ikivugoto) | 0 | 42.50 | 297.0 |
| Heat treated | 0 | 9.37 | 97.5 |
| Fermented by industrial processes | 0 | 18.60 | 142.0 |
| Other dairy products | 0 | 13.50 | 116.0 |

## YLL

| Food | 2.5% | Mean | 97.5% |
| --- | --- | --- | --- |
| DAIRY | 0 | 179.00 | 1100.0 |
| Milk from cattle | 0 | 159.00 | 1020.0 |
| Milk from other animals | 0 | 19.80 | 181.0 |
| Consumed raw | 0 | 77.20 | 519.0 |
| Fermented by traditional processes (e.g. ikivugoto) | 0 | 41.60 | 292.0 |
| Heat treated | 0 | 9.16 | 95.9 |
| Fermented by industrial processes | 0 | 18.20 | 138.0 |
| Other dairy products | 0 | 13.20 | 114.0 |

## YLD

| Food | 2.5% | Mean | 97.5% |
| --- | --- | --- | --- |
| DAIRY | 0 | 4.080 | 27.10 |
| Milk from cattle | 0 | 3.630 | 23.90 |
| Milk from other animals | 0 | 0.450 | 4.19 |
| Consumed raw | 0 | 1.760 | 12.40 |
| Fermented by traditional processes (e.g. ikivugoto) | 0 | 0.953 | 7.28 |
| Heat treated | 0 | 0.213 | 2.08 |
| Fermented by industrial processes | 0 | 0.413 | 3.24 |
| Other dairy products | 0 | 0.296 | 2.66 |

## Incidence rate (per 100,000 population)

| Food | 2.5% | Mean | 97.5% |
| --- | --- | --- | --- |
| DAIRY | 0 | 205.0 | 1360 |
| Milk from cattle | 0 | 183.0 | 1230 |
| Milk from other animals | 0 | 22.5 | 212 |
| Consumed raw | 0 | 88.0 | 634 |
| Fermented by traditional processes (e.g. ikivugoto) | 0 | 48.0 | 358 |
| Heat treated | 0 | 10.7 | 103 |
| Fermented by industrial processes | 0 | 20.8 | 164 |
| Other dairy products | 0 | 14.9 | 135 |

## Mortality rate (per 100,000 population)

| Food | 2.5% | Mean | 97.5% |
| --- | --- | --- | --- |
| DAIRY | 0 | 0.11200 | 0.6880 |
| Milk from cattle | 0 | 0.09960 | 0.6350 |
| Milk from other animals | 0 | 0.01240 | 0.1130 |
| Consumed raw | 0 | 0.04830 | 0.3250 |
| Fermented by traditional processes (e.g. ikivugoto) | 0 | 0.02600 | 0.1830 |
| Heat treated | 0 | 0.00573 | 0.0599 |
| Fermented by industrial processes | 0 | 0.01140 | 0.0865 |
| Other dairy products | 0 | 0.00825 | 0.0713 |

## DALY rate (per 100,000 population)

| Food | 2.5% | Mean | 97.5% |
| --- | --- | --- | --- |
| DAIRY | 0 | 10.300 | 62.60 |
| Milk from cattle | 0 | 9.130 | 57.80 |
| Milk from other animals | 0 | 1.130 | 10.40 |
| Consumed raw | 0 | 4.420 | 29.70 |
| Fermented by traditional processes (e.g. ikivugoto) | 0 | 2.380 | 16.60 |
| Heat treated | 0 | 0.525 | 5.46 |
| Fermented by industrial processes | 0 | 1.040 | 7.96 |
| Other dairy products | 0 | 0.756 | 6.50 |

## YLL rate (per 100,000 population)

| Food | 2.5% | Mean | 97.5% |
| --- | --- | --- | --- |
| DAIRY | 0 | 10.000 | 61.60 |
| Milk from cattle | 0 | 8.930 | 56.90 |
| Milk from other animals | 0 | 1.110 | 10.20 |
| Consumed raw | 0 | 4.330 | 29.10 |
| Fermented by traditional processes (e.g. ikivugoto) | 0 | 2.330 | 16.40 |
| Heat treated | 0 | 0.513 | 5.37 |
| Fermented by industrial processes | 0 | 1.020 | 7.75 |
| Other dairy products | 0 | 0.739 | 6.39 |

## YLD rate (per 100,000 population)

| Food | 2.5% | Mean | 97.5% |
| --- | --- | --- | --- |
| DAIRY | 0 | 0.2290 | 1.520 |
| Milk from cattle | 0 | 0.2030 | 1.340 |
| Milk from other animals | 0 | 0.0252 | 0.234 |
| Consumed raw | 0 | 0.0984 | 0.697 |
| Fermented by traditional processes (e.g. ikivugoto) | 0 | 0.0534 | 0.408 |
| Heat treated | 0 | 0.0119 | 0.116 |
| Fermented by industrial processes | 0 | 0.0231 | 0.181 |
| Other dairy products | 0 | 0.0166 | 0.149 |

```

Children over the age of 5 and adults

## Incidence

| Food | 2.5% | Mean | 97.5% |
| --- | --- | --- | --- |
| DAIRY | 0 | 806.0 | 6220 |
| Milk from cattle | 0 | 717.0 | 5700 |
| Milk from other animals | 0 | 89.1 | 739 |
| Consumed raw | 0 | 348.0 | 2730 |
| Fermented by traditional processes (e.g. ikivugoto) | 0 | 189.0 | 1530 |
| Heat treated | 0 | 40.8 | 335 |
| Fermented by industrial processes | 0 | 82.9 | 620 |
| Other dairy products | 0 | 56.0 | 510 |

## Mortality

| Food | 2.5% | Mean | 97.5% |
| --- | --- | --- | --- |
| DAIRY | 0 | 1.0600 | 6.090 |
| Milk from cattle | 0 | 0.9480 | 5.620 |
| Milk from other animals | 0 | 0.1170 | 1.060 |
| Consumed raw | 0 | 0.4600 | 2.920 |
| Fermented by traditional processes (e.g. ikivugoto) | 0 | 0.2470 | 1.650 |
| Heat treated | 0 | 0.0546 | 0.558 |
| Fermented by industrial processes | 0 | 0.1080 | 0.831 |
| Other dairy products | 0 | 0.0779 | 0.665 |

## DALY

| Food | 2.5% | Mean | 97.5% |
| --- | --- | --- | --- |
| DAIRY | 0 | 71.10 | 405.0 |
| Milk from cattle | 0 | 63.30 | 374.0 |
| Milk from other animals | 0 | 7.79 | 70.5 |
| Consumed raw | 0 | 30.70 | 196.0 |
| Fermented by traditional processes (e.g. ikivugoto) | 0 | 16.50 | 111.0 |
| Heat treated | 0 | 3.65 | 37.5 |
| Fermented by industrial processes | 0 | 7.22 | 55.3 |
| Other dairy products | 0 | 5.20 | 44.3 |

## YLL

| Food | 2.5% | Mean | 97.5% |
| --- | --- | --- | --- |
| DAIRY | 0 | 70.60 | 404.0 |
| Milk from cattle | 0 | 62.90 | 373.0 |
| Milk from other animals | 0 | 7.74 | 70.2 |
| Consumed raw | 0 | 30.50 | 194.0 |
| Fermented by traditional processes (e.g. ikivugoto) | 0 | 16.40 | 109.0 |
| Heat treated | 0 | 3.62 | 37.0 |
| Fermented by industrial processes | 0 | 7.17 | 55.1 |
| Other dairy products | 0 | 5.17 | 44.1 |

## YLD

| Food | 2.5% | Mean | 97.5% |
| --- | --- | --- | --- |
| DAIRY | 0 | 0.4580 | 3.540 |
| Milk from cattle | 0 | 0.4080 | 3.240 |
| Milk from other animals | 0 | 0.0506 | 0.420 |
| Consumed raw | 0 | 0.1980 | 1.550 |
| Fermented by traditional processes (e.g. ikivugoto) | 0 | 0.1070 | 0.871 |
| Heat treated | 0 | 0.0232 | 0.190 |
| Fermented by industrial processes | 0 | 0.0471 | 0.352 |
| Other dairy products | 0 | 0.0318 | 0.290 |

## Incidence rate (per 100,000 population)

| Food | 2.5% | Mean | 97.5% |
| --- | --- | --- | --- |
| DAIRY | 0 | 8.910 | 68.70 |
| Milk from cattle | 0 | 7.920 | 63.00 |
| Milk from other animals | 0 | 0.984 | 8.17 |
| Consumed raw | 0 | 3.850 | 30.10 |
| Fermented by traditional processes (e.g. ikivugoto) | 0 | 2.090 | 16.90 |
| Heat treated | 0 | 0.451 | 3.70 |
| Fermented by industrial processes | 0 | 0.916 | 6.85 |
| Other dairy products | 0 | 0.618 | 5.64 |

## Mortality rate (per 100,000 population)

| Food | 2.5% | Mean | 97.5% |
| --- | --- | --- | --- |
| DAIRY | 0 | 0.011800 | 0.06730 |
| Milk from cattle | 0 | 0.010500 | 0.06210 |
| Milk from other animals | 0 | 0.001290 | 0.01170 |
| Consumed raw | 0 | 0.005090 | 0.03220 |
| Fermented by traditional processes (e.g. ikivugoto) | 0 | 0.002730 | 0.01820 |
| Heat treated | 0 | 0.000603 | 0.00617 |
| Fermented by industrial processes | 0 | 0.001190 | 0.00918 |
| Other dairy products | 0 | 0.000861 | 0.00735 |

## DALY rate (per 100,000 population)

| Food | 2.5% | Mean | 97.5% |
| --- | --- | --- | --- |
| DAIRY | 0 | 0.7860 | 4.480 |
| Milk from cattle | 0 | 0.7000 | 4.140 |
| Milk from other animals | 0 | 0.0860 | 0.779 |
| Consumed raw | 0 | 0.3400 | 2.160 |
| Fermented by traditional processes (e.g. ikivugoto) | 0 | 0.1820 | 1.220 |
| Heat treated | 0 | 0.0403 | 0.415 |
| Fermented by industrial processes | 0 | 0.0797 | 0.611 |
| Other dairy products | 0 | 0.0574 | 0.489 |

## YLL rate (per 100,000 population)

| Food | 2.5% | Mean | 97.5% |
| --- | --- | --- | --- |
| DAIRY | 0 | 0.7810 | 4.460 |
| Milk from cattle | 0 | 0.6950 | 4.120 |
| Milk from other animals | 0 | 0.0855 | 0.775 |
| Consumed raw | 0 | 0.3370 | 2.140 |
| Fermented by traditional processes (e.g. ikivugoto) | 0 | 0.1810 | 1.210 |
| Heat treated | 0 | 0.0400 | 0.409 |
| Fermented by industrial processes | 0 | 0.0792 | 0.609 |
| Other dairy products | 0 | 0.0571 | 0.487 |

## YLD rate (per 100,000 population)

| Food | 2.5% | Mean | 97.5% |
| --- | --- | --- | --- |
| DAIRY | 0 | 0.005060 | 0.03910 |
| Milk from cattle | 0 | 0.004500 | 0.03580 |
| Milk from other animals | 0 | 0.000559 | 0.00464 |
| Consumed raw | 0 | 0.002190 | 0.01710 |
| Fermented by traditional processes (e.g. ikivugoto) | 0 | 0.001190 | 0.00962 |
| Heat treated | 0 | 0.000256 | 0.00210 |
| Fermented by industrial processes | 0 | 0.000520 | 0.00389 |
| Other dairy products | 0 | 0.000351 | 0.00320 |

```
