# Supplementary material for: Estimates of disease burden caused by foodborne pathogens in contaminated dairy products in Rwanda
Source: BMC Public Health. 2023 Apr 6;23:657. doi: 10.1186/s12889-023-15204-x (PMC10077627; doi:10.1186/s12889-023-15204-x)
Supplement: Supplementary file 4 — Supplementary Material 4 [file 12889_2023_15204_MOESM4_ESM.docx]

Burden of *Mycobacterium bovis* in dairy; Rwanda, 2010

Total population

## Incidence

| Food | 2.5% | Mean | 97.5% |
| --- | --- | --- | --- |
| DAIRY | 174.000000 | 267.00 | 347.0 |
| Milk from cattle | 129.000000 | 250.00 | 341.0 |
| Milk from other animals | 0.009450 | 16.10 | 135.0 |
| Consumed raw | 46.100000 | 169.00 | 281.0 |
| Fermented by traditional processes (e.g. ikivugoto) | 2.290000 | 42.70 | 141.0 |
| Heat treated | 0.008290 | 14.20 | 113.0 |
| Fermented by industrial processes | 0.000263 | 15.40 | 89.9 |
| Other dairy products | 0.640000 | 9.71 | 54.6 |

## Mortality

| Food | 2.5% | Mean | 97.5% |
| --- | --- | --- | --- |
| DAIRY | 10.9000000 | 24.800 | 45.30 |
| Milk from cattle | 8.9700000 | 23.300 | 44.00 |
| Milk from other animals | 0.0008260 | 1.490 | 12.50 |
| Consumed raw | 3.4700000 | 15.700 | 33.00 |
| Fermented by traditional processes (e.g. ikivugoto) | 0.1940000 | 3.970 | 14.30 |
| Heat treated | 0.0007110 | 1.320 | 10.30 |
| Fermented by industrial processes | 0.0000251 | 1.430 | 8.39 |
| Other dairy products | 0.0566000 | 0.902 | 4.74 |

##

## DALY

| Food | 2.5% | Mean | 97.5% |
| --- | --- | --- | --- |
| DAIRY | 657.00000 | 1410.0 | 2510 |
| Milk from cattle | 537.00000 | 1330.0 | 2440 |
| Milk from other animals | 0.04680 | 84.7 | 706 |
| Consumed raw | 196.00000 | 892.0 | 1840 |
| Fermented by traditional processes (e.g. ikivugoto) | 11.10000 | 225.0 | 802 |
| Heat treated | 0.04060 | 74.8 | 581 |
| Fermented by industrial processes | 0.00145 | 81.5 | 477 |
| Other dairy products | 3.32000 | 51.3 | 272 |

## YLL

| Food | 2.5% | Mean | 97.5% |
| --- | --- | --- | --- |
| DAIRY | 582.00000 | 1320.0 | 2410 |
| Milk from cattle | 478.00000 | 1240.0 | 2350 |
| Milk from other animals | 0.04400 | 79.4 | 664 |
| Consumed raw | 185.00000 | 837.0 | 1760 |
| Fermented by traditional processes (e.g. ikivugoto) | 10.30000 | 211.0 | 759 |
| Heat treated | 0.03790 | 70.2 | 548 |
| Fermented by industrial processes | 0.00134 | 76.4 | 447 |
| Other dairy products | 3.02000 | 48.0 | 253 |

## YLD

| Food | 2.5% | Mean | 97.5% |
| --- | --- | --- | --- |
| DAIRY | 57.5000000 | 88.20 | 115.0 |
| Milk from cattle | 42.9000000 | 82.90 | 113.0 |
| Milk from other animals | 0.0031300 | 5.33 | 44.8 |
| Consumed raw | 15.2000000 | 55.80 | 92.9 |
| Fermented by traditional processes (e.g. ikivugoto) | 0.7590000 | 14.10 | 46.5 |
| Heat treated | 0.0027400 | 4.69 | 37.5 |
| Fermented by industrial processes | 0.0000872 | 5.08 | 29.8 |
| Other dairy products | 0.2120000 | 3.21 | 18.1 |

##

## Incidence rate (per 100,000 population)

| Food | 2.5% | Mean | 97.5% |
| --- | --- | --- | --- |
| DAIRY | 1.6000000 | 2.4600 | 3.200 |
| Milk from cattle | 1.1900000 | 2.3100 | 3.150 |
| Milk from other animals | 0.0000872 | 0.1490 | 1.250 |
| Consumed raw | 0.4250000 | 1.5600 | 2.590 |
| Fermented by traditional processes (e.g. ikivugoto) | 0.0212000 | 0.3940 | 1.300 |
| Heat treated | 0.0000765 | 0.1310 | 1.050 |
| Fermented by industrial processes | 0.0000024 | 0.1420 | 0.830 |
| Other dairy products | 0.0059000 | 0.0896 | 0.504 |

## Mortality rate (per 100,000 population)

| Food | 2.5% | Mean | 97.5% |
| --- | --- | --- | --- |
| DAIRY | 0.1010000 | 0.22900 | 0.4180 |
| Milk from cattle | 0.0828000 | 0.21500 | 0.4060 |
| Milk from other animals | 0.0000076 | 0.01370 | 0.1150 |
| Consumed raw | 0.0320000 | 0.14500 | 0.3040 |
| Fermented by traditional processes (e.g. ikivugoto) | 0.0017900 | 0.03660 | 0.1320 |
| Heat treated | 0.0000066 | 0.01210 | 0.0949 |
| Fermented by industrial processes | 0.0000002 | 0.01320 | 0.0774 |
| Other dairy products | 0.0005230 | 0.00832 | 0.0438 |

##

## DALY rate (per 100,000 population)

| Food | 2.5% | Mean | 97.5% |
| --- | --- | --- | --- |
| DAIRY | 6.0700000 | 13.000 | 23.20 |
| Milk from cattle | 4.9500000 | 12.200 | 22.50 |
| Milk from other animals | 0.0004320 | 0.782 | 6.51 |
| Consumed raw | 1.8100000 | 8.230 | 17.00 |
| Fermented by traditional processes (e.g. ikivugoto) | 0.1020000 | 2.080 | 7.40 |
| Heat treated | 0.0003740 | 0.691 | 5.36 |
| Fermented by industrial processes | 0.0000134 | 0.752 | 4.40 |
| Other dairy products | 0.0307000 | 0.473 | 2.51 |

## YLL rate (per 100,000 population)

| Food | 2.5% | Mean | 97.5% |
| --- | --- | --- | --- |
| DAIRY | 5.3700000 | 12.200 | 22.30 |
| Milk from cattle | 4.4100000 | 11.500 | 21.70 |
| Milk from other animals | 0.0004060 | 0.733 | 6.13 |
| Consumed raw | 1.7100000 | 7.720 | 16.20 |
| Fermented by traditional processes (e.g. ikivugoto) | 0.0955000 | 1.950 | 7.01 |
| Heat treated | 0.0003500 | 0.647 | 5.06 |
| Fermented by industrial processes | 0.0000123 | 0.705 | 4.13 |
| Other dairy products | 0.0279000 | 0.443 | 2.33 |

## YLD rate (per 100,000 population)

| Food | 2.5% | Mean | 97.5% |
| --- | --- | --- | --- |
| DAIRY | 0.5310000 | 0.8140 | 1.060 |
| Milk from cattle | 0.3950000 | 0.7650 | 1.040 |
| Milk from other animals | 0.0000289 | 0.0492 | 0.413 |
| Consumed raw | 0.1410000 | 0.5150 | 0.857 |
| Fermented by traditional processes (e.g. ikivugoto) | 0.0070100 | 0.1310 | 0.429 |
| Heat treated | 0.0000253 | 0.0433 | 0.346 |
| Fermented by industrial processes | 0.0000008 | 0.0469 | 0.275 |
| Other dairy products | 0.0019500 | 0.0296 | 0.167 |

Children under 5 years of age

## Incidence

| Food | 2.5% | Mean | 97.5% |
| --- | --- | --- | --- |
| DAIRY | 1.2800000 | 1.9700 | 2.560 |
| Milk from cattle | 0.9550000 | 1.8500 | 2.510 |
| Milk from other animals | 0.0000697 | 0.1190 | 0.998 |
| Consumed raw | 0.3400000 | 1.2400 | 2.070 |
| Fermented by traditional processes (e.g. ikivugoto) | 0.0169000 | 0.3150 | 1.040 |
| Heat treated | 0.0000611 | 0.1050 | 0.837 |
| Fermented by industrial processes | 0.0000019 | 0.1130 | 0.663 |
| Other dairy products | 0.0047200 | 0.0716 | 0.403 |

## Mortality

| Food | 2.5% | Mean | 97.5% |
| --- | --- | --- | --- |
| DAIRY | 0.0805000 | 0.18300 | 0.3340 |
| Milk from cattle | 0.0662000 | 0.17200 | 0.3250 |
| Milk from other animals | 0.0000061 | 0.01100 | 0.0919 |
| Consumed raw | 0.0256000 | 0.11600 | 0.2430 |
| Fermented by traditional processes (e.g. ikivugoto) | 0.0014300 | 0.02920 | 0.1050 |
| Heat treated | 0.0000052 | 0.00971 | 0.0759 |
| Fermented by industrial processes | 0.0000002 | 0.01060 | 0.0619 |
| Other dairy products | 0.0004180 | 0.00665 | 0.0350 |

## DALY

| Food | 2.5% | Mean | 97.5% |
| --- | --- | --- | --- |
| DAIRY | 7.7600000 | 17.000 | 30.60 |
| Milk from cattle | 6.3800000 | 16.000 | 29.80 |
| Milk from other animals | 0.0005660 | 1.020 | 8.52 |
| Consumed raw | 2.3800000 | 10.800 | 22.40 |
| Fermented by traditional processes (e.g. ikivugoto) | 0.1340000 | 2.720 | 9.72 |
| Heat treated | 0.0004880 | 0.905 | 7.03 |
| Fermented by industrial processes | 0.0000174 | 0.985 | 5.75 |
| Other dairy products | 0.0395000 | 0.619 | 3.28 |

## YLL

| Food | 2.5% | Mean | 97.5% |
| --- | --- | --- | --- |
| DAIRY | 7.2200000 | 16.400 | 29.90 |
| Milk from cattle | 5.9300000 | 15.400 | 29.10 |
| Milk from other animals | 0.0005460 | 0.985 | 8.24 |
| Consumed raw | 2.2900000 | 10.400 | 21.80 |
| Fermented by traditional processes (e.g. ikivugoto) | 0.1280000 | 2.620 | 9.42 |
| Heat treated | 0.0004700 | 0.870 | 6.80 |
| Fermented by industrial processes | 0.0000166 | 0.947 | 5.54 |
| Other dairy products | 0.0374000 | 0.596 | 3.13 |

## YLD

| Food | 2.5% | Mean | 97.5% |
| --- | --- | --- | --- |
| DAIRY | 0.4240000 | 0.6510 | 0.847 |
| Milk from cattle | 0.3160000 | 0.6110 | 0.832 |
| Milk from other animals | 0.0000231 | 0.0393 | 0.330 |
| Consumed raw | 0.1120000 | 0.4110 | 0.685 |
| Fermented by traditional processes (e.g. ikivugoto) | 0.0056000 | 0.1040 | 0.343 |
| Heat treated | 0.0000202 | 0.0346 | 0.277 |
| Fermented by industrial processes | 0.0000006 | 0.0375 | 0.219 |
| Other dairy products | 0.0015600 | 0.0237 | 0.133 |

## Incidence rate (per 100,000 population)

| Food | 2.5% | Mean | 97.5% |
| --- | --- | --- | --- |
| DAIRY | 0.0717000 | 0.11000 | 0.1430 |
| Milk from cattle | 0.0535000 | 0.10300 | 0.1410 |
| Milk from other animals | 0.0000039 | 0.00665 | 0.0559 |
| Consumed raw | 0.0190000 | 0.06960 | 0.1160 |
| Fermented by traditional processes (e.g. ikivugoto) | 0.0009470 | 0.01770 | 0.0581 |
| Heat treated | 0.0000034 | 0.00585 | 0.0468 |
| Fermented by industrial processes | 0.0000001 | 0.00634 | 0.0371 |
| Other dairy products | 0.0002640 | 0.00401 | 0.0225 |

## Mortality rate (per 100,000 population)

| Food | 2.5% | Mean | 97.5% |
| --- | --- | --- | --- |
| DAIRY | 0.0045100 | 0.010200 | 0.01870 |
| Milk from cattle | 0.0037000 | 0.009630 | 0.01820 |
| Milk from other animals | 0.0000003 | 0.000615 | 0.00515 |
| Consumed raw | 0.0014300 | 0.006480 | 0.01360 |
| Fermented by traditional processes (e.g. ikivugoto) | 0.0000802 | 0.001640 | 0.00589 |
| Heat treated | 0.0000003 | 0.000544 | 0.00425 |
| Fermented by industrial processes | 0.0000000 | 0.000592 | 0.00346 |
| Other dairy products | 0.0000234 | 0.000372 | 0.00196 |

## DALY rate (per 100,000 population)

| Food | 2.5% | Mean | 97.5% |
| --- | --- | --- | --- |
| DAIRY | 0.4350000 | 0.9540 | 1.720 |
| Milk from cattle | 0.3570000 | 0.8970 | 1.670 |
| Milk from other animals | 0.0000317 | 0.0573 | 0.477 |
| Consumed raw | 0.1330000 | 0.6040 | 1.250 |
| Fermented by traditional processes (e.g. ikivugoto) | 0.0075000 | 0.1530 | 0.544 |
| Heat treated | 0.0000274 | 0.0507 | 0.394 |
| Fermented by industrial processes | 0.0000010 | 0.0551 | 0.322 |
| Other dairy products | 0.0022100 | 0.0347 | 0.183 |

## YLL rate (per 100,000 population)

| Food | 2.5% | Mean | 97.5% |
| --- | --- | --- | --- |
| DAIRY | 0.4040000 | 0.9180 | 1.670 |
| Milk from cattle | 0.3320000 | 0.8630 | 1.630 |
| Milk from other animals | 0.0000306 | 0.0551 | 0.461 |
| Consumed raw | 0.1280000 | 0.5810 | 1.220 |
| Fermented by traditional processes (e.g. ikivugoto) | 0.0071800 | 0.1470 | 0.527 |
| Heat treated | 0.0000263 | 0.0487 | 0.381 |
| Fermented by industrial processes | 0.0000009 | 0.0530 | 0.310 |
| Other dairy products | 0.0021000 | 0.0334 | 0.175 |

## YLD rate (per 100,000 population)

| Food | 2.5% | Mean | 97.5% |
| --- | --- | --- | --- |
| DAIRY | 0.0237000 | 0.03640 | 0.04740 |
| Milk from cattle | 0.0177000 | 0.03420 | 0.04660 |
| Milk from other animals | 0.0000013 | 0.00220 | 0.01850 |
| Consumed raw | 0.0063000 | 0.02300 | 0.03840 |
| Fermented by traditional processes (e.g. ikivugoto) | 0.0003140 | 0.00584 | 0.01920 |
| Heat treated | 0.0000011 | 0.00194 | 0.01550 |
| Fermented by industrial processes | 0.0000000 | 0.00210 | 0.01230 |
| Other dairy products | 0.0000875 | 0.00133 | 0.00746 |

```

Children over 5 years of age and adults

## Incidence

| Food | 2.5% | Mean | 97.5% |
| --- | --- | --- | --- |
| DAIRY | 172.000000 | 265.00 | 344.0 |
| Milk from cattle | 129.000000 | 249.00 | 338.0 |
| Milk from other animals | 0.009380 | 16.00 | 134.0 |
| Consumed raw | 45.700000 | 167.00 | 279.0 |
| Fermented by traditional processes (e.g. ikivugoto) | 2.280000 | 42.40 | 140.0 |
| Heat treated | 0.008230 | 14.10 | 113.0 |
| Fermented by industrial processes | 0.000261 | 15.20 | 89.2 |
| Other dairy products | 0.635000 | 9.63 | 54.2 |

## Mortality

| Food | 2.5% | Mean | 97.5% |
| --- | --- | --- | --- |
| DAIRY | 10.8000000 | 24.600 | 44.90 |
| Milk from cattle | 8.9000000 | 23.100 | 43.70 |
| Milk from other animals | 0.0008200 | 1.480 | 12.40 |
| Consumed raw | 3.4400000 | 15.600 | 32.70 |
| Fermented by traditional processes (e.g. ikivugoto) | 0.1930000 | 3.940 | 14.10 |
| Heat treated | 0.0007060 | 1.310 | 10.20 |
| Fermented by industrial processes | 0.0000249 | 1.420 | 8.33 |
| Other dairy products | 0.0562000 | 0.895 | 4.71 |

## DALY

| Food | 2.5% | Mean | 97.5% |
| --- | --- | --- | --- |
| DAIRY | 650.00000 | 1390.0 | 2480 |
| Milk from cattle | 531.00000 | 1310.0 | 2410 |
| Milk from other animals | 0.04620 | 83.7 | 697 |
| Consumed raw | 194.00000 | 882.0 | 1820 |
| Fermented by traditional processes (e.g. ikivugoto) | 11.00000 | 223.0 | 792 |
| Heat treated | 0.04010 | 73.9 | 574 |
| Fermented by industrial processes | 0.00143 | 80.5 | 472 |
| Other dairy products | 3.28000 | 50.6 | 269 |

## YLL

| Food | 2.5% | Mean | 97.5% |
| --- | --- | --- | --- |
| DAIRY | 575.00000 | 1310.0 | 2380 |
| Milk from cattle | 472.00000 | 1230.0 | 2320 |
| Milk from other animals | 0.04350 | 78.4 | 656 |
| Consumed raw | 183.00000 | 826.0 | 1730 |
| Fermented by traditional processes (e.g. ikivugoto) | 10.20000 | 209.0 | 750 |
| Heat treated | 0.03740 | 69.3 | 541 |
| Fermented by industrial processes | 0.00132 | 75.4 | 442 |
| Other dairy products | 2.98000 | 47.4 | 250 |

## YLD

| Food | 2.5% | Mean | 97.5% |
| --- | --- | --- | --- |
| DAIRY | 57.1000000 | 87.60 | 114.0 |
| Milk from cattle | 42.5000000 | 82.30 | 112.0 |
| Milk from other animals | 0.0031100 | 5.29 | 44.5 |
| Consumed raw | 15.1000000 | 55.40 | 92.2 |
| Fermented by traditional processes (e.g. ikivugoto) | 0.7540000 | 14.00 | 46.2 |
| Heat treated | 0.0027200 | 4.66 | 37.3 |
| Fermented by industrial processes | 0.0000865 | 5.04 | 29.5 |
| Other dairy products | 0.2100000 | 3.19 | 17.9 |

## Incidence rate (per 100,000 population)

| Food | 2.5% | Mean | 97.5% |
| --- | --- | --- | --- |
| DAIRY | 1.9000000 | 2.920 | 3.800 |
| Milk from cattle | 1.4200000 | 2.750 | 3.740 |
| Milk from other animals | 0.0001040 | 0.177 | 1.480 |
| Consumed raw | 0.5050000 | 1.850 | 3.080 |
| Fermented by traditional processes (e.g. ikivugoto) | 0.0252000 | 0.469 | 1.540 |
| Heat treated | 0.0000909 | 0.155 | 1.240 |
| Fermented by industrial processes | 0.0000029 | 0.168 | 0.986 |
| Other dairy products | 0.0070200 | 0.106 | 0.599 |

## Mortality rate (per 100,000 population)

| Food | 2.5% | Mean | 97.5% |
| --- | --- | --- | --- |
| DAIRY | 0.1200000 | 0.27200 | 0.496 |
| Milk from cattle | 0.0984000 | 0.25600 | 0.483 |
| Milk from other animals | 0.0000091 | 0.01630 | 0.137 |
| Consumed raw | 0.0380000 | 0.17200 | 0.362 |
| Fermented by traditional processes (e.g. ikivugoto) | 0.0021300 | 0.04350 | 0.156 |
| Heat treated | 0.0000078 | 0.01440 | 0.113 |
| Fermented by industrial processes | 0.0000003 | 0.01570 | 0.092 |
| Other dairy products | 0.0006210 | 0.00989 | 0.052 |

## DALY rate (per 100,000 population)

| Food | 2.5% | Mean | 97.5% |
| --- | --- | --- | --- |
| DAIRY | 7.1800000 | 15.400 | 27.40 |
| Milk from cattle | 5.8600000 | 14.500 | 26.60 |
| Milk from other animals | 0.0005110 | 0.925 | 7.71 |
| Consumed raw | 2.1400000 | 9.740 | 20.10 |
| Fermented by traditional processes (e.g. ikivugoto) | 0.1210000 | 2.460 | 8.75 |
| Heat treated | 0.0004430 | 0.817 | 6.34 |
| Fermented by industrial processes | 0.0000158 | 0.889 | 5.21 |
| Other dairy products | 0.0363000 | 0.559 | 2.97 |

## YLL rate (per 100,000 population)

| Food | 2.5% | Mean | 97.5% |
| --- | --- | --- | --- |
| DAIRY | 6.3500000 | 14.400 | 26.30 |
| Milk from cattle | 5.2200000 | 13.600 | 25.60 |
| Milk from other animals | 0.0004800 | 0.866 | 7.25 |
| Consumed raw | 2.0200000 | 9.130 | 19.20 |
| Fermented by traditional processes (e.g. ikivugoto) | 0.1130000 | 2.310 | 8.29 |
| Heat treated | 0.0004130 | 0.765 | 5.98 |
| Fermented by industrial processes | 0.0000146 | 0.833 | 4.88 |
| Other dairy products | 0.0329000 | 0.524 | 2.76 |

## YLD rate (per 100,000 population)

| Food | 2.5% | Mean | 97.5% |
| --- | --- | --- | --- |
| DAIRY | 0.6310000 | 0.9680 | 1.260 |
| Milk from cattle | 0.4700000 | 0.9090 | 1.240 |
| Milk from other animals | 0.0000343 | 0.0585 | 0.491 |
| Consumed raw | 0.1670000 | 0.6120 | 1.020 |
| Fermented by traditional processes (e.g. ikivugoto) | 0.0083300 | 0.1550 | 0.510 |
| Heat treated | 0.0000301 | 0.0515 | 0.412 |
| Fermented by industrial processes | 0.0000010 | 0.0557 | 0.326 |
| Other dairy products | 0.0023200 | 0.0352 | 0.198 |

```
