# Supplementary material for: Estimates of disease burden caused by foodborne pathogens in contaminated dairy products in Rwanda
Source: BMC Public Health. 2023 Apr 6;23:657. doi: 10.1186/s12889-023-15204-x (PMC10077627; doi:10.1186/s12889-023-15204-x)
Supplement: Supplementary file 5 — Supplementary Material 5 [file 12889_2023_15204_MOESM5_ESM.docx]

Burden of non-typhoidal *Salmonella enterica* in dairy; Rwanda, 2010

Total population

## Incidence

| Food | 2.5% | Mean | 97.5% |
| --- | --- | --- | --- |
| DAIRY | 0 | 7560 | 31500 |
| Milk from cattle | 0 | 7110 | 29800 |
| Milk from other animals | 0 | 447 | 4100 |
| Consumed raw | 0 | 3480 | 15500 |
| Fermented by traditional processes (e.g. ikivugoto) | 0 | 1640 | 7880 |
| Heat treated | 0 | 431 | 3550 |
| Fermented by industrial processes | 0 | 1170 | 6230 |
| Other dairy products | 0 | 392 | 2640 |

## Mortality

| Food | 2.5% | Mean | 97.5% |
| --- | --- | --- | --- |
| DAIRY | 0 | 11.300 | 37.00 |
| Milk from cattle | 0 | 10.700 | 35.50 |
| Milk from other animals | 0 | 0.672 | 6.34 |
| Consumed raw | 0 | 5.210 | 19.00 |
| Fermented by traditional processes (e.g. ikivugoto) | 0 | 2.450 | 9.73 |
| Heat treated | 0 | 0.655 | 5.25 |
| Fermented by industrial processes | 0 | 1.750 | 7.75 |
| Other dairy products | 0 | 0.593 | 3.90 |

##

## DALY

| Food | 2.5% | Mean | 97.5% |
| --- | --- | --- | --- |
| DAIRY | 0 | 853.0 | 2780 |
| Milk from cattle | 0 | 802.0 | 2680 |
| Milk from other animals | 0 | 50.6 | 474 |
| Consumed raw | 0 | 392.0 | 1430 |
| Fermented by traditional processes (e.g. ikivugoto) | 0 | 184.0 | 731 |
| Heat treated | 0 | 49.3 | 395 |
| Fermented by industrial processes | 0 | 132.0 | 584 |
| Other dairy products | 0 | 44.6 | 292 |

## YLL

| Food | 2.5% | Mean | 97.5% |
| --- | --- | --- | --- |
| DAIRY | 0 | 844.0 | 2750 |
| Milk from cattle | 0 | 794.0 | 2660 |
| Milk from other animals | 0 | 50.1 | 472 |
| Consumed raw | 0 | 388.0 | 1420 |
| Fermented by traditional processes (e.g. ikivugoto) | 0 | 182.0 | 724 |
| Heat treated | 0 | 48.8 | 390 |
| Fermented by industrial processes | 0 | 131.0 | 579 |
| Other dairy products | 0 | 44.2 | 290 |

## YLD

| Food | 2.5% | Mean | 97.5% |
| --- | --- | --- | --- |
| DAIRY | 0 | 8.780 | 36.60 |
| Milk from cattle | 0 | 8.260 | 34.50 |
| Milk from other animals | 0 | 0.522 | 4.74 |
| Consumed raw | 0 | 4.040 | 18.00 |
| Fermented by traditional processes (e.g. ikivugoto) | 0 | 1.910 | 9.27 |
| Heat treated | 0 | 0.499 | 4.08 |
| Fermented by industrial processes | 0 | 1.360 | 7.14 |
| Other dairy products | 0 | 0.452 | 3.05 |

##

## Incidence rate (per 100,000 population)

| Food | 2.5% | Mean | 97.5% |
| --- | --- | --- | --- |
| DAIRY | 0 | 69.70 | 291.0 |
| Milk from cattle | 0 | 65.60 | 275.0 |
| Milk from other animals | 0 | 4.12 | 37.8 |
| Consumed raw | 0 | 32.10 | 143.0 |
| Fermented by traditional processes (e.g. ikivugoto) | 0 | 15.10 | 72.7 |
| Heat treated | 0 | 3.97 | 32.7 |
| Fermented by industrial processes | 0 | 10.80 | 57.5 |
| Other dairy products | 0 | 3.61 | 24.3 |

## Mortality rate (per 100,000 population)

| Food | 2.5% | Mean | 97.5% |
| --- | --- | --- | --- |
| DAIRY | 0 | 0.10500 | 0.3410 |
| Milk from cattle | 0 | 0.09830 | 0.3280 |
| Milk from other animals | 0 | 0.00620 | 0.0585 |
| Consumed raw | 0 | 0.04800 | 0.1750 |
| Fermented by traditional processes (e.g. ikivugoto) | 0 | 0.02260 | 0.0898 |
| Heat treated | 0 | 0.00604 | 0.0485 |
| Fermented by industrial processes | 0 | 0.01620 | 0.0715 |
| Other dairy products | 0 | 0.00547 | 0.0360 |

##

## DALY rate (per 100,000 population)

| Food | 2.5% | Mean | 97.5% |
| --- | --- | --- | --- |
| DAIRY | 0 | 7.870 | 25.70 |
| Milk from cattle | 0 | 7.400 | 24.70 |
| Milk from other animals | 0 | 0.467 | 4.37 |
| Consumed raw | 0 | 3.620 | 13.20 |
| Fermented by traditional processes (e.g. ikivugoto) | 0 | 1.700 | 6.74 |
| Heat treated | 0 | 0.455 | 3.65 |
| Fermented by industrial processes | 0 | 1.220 | 5.39 |
| Other dairy products | 0 | 0.412 | 2.69 |

## YLL rate (per 100,000 population)

| Food | 2.5% | Mean | 97.5% |
| --- | --- | --- | --- |
| DAIRY | 0 | 7.790 | 25.40 |
| Milk from cattle | 0 | 7.330 | 24.50 |
| Milk from other animals | 0 | 0.462 | 4.36 |
| Consumed raw | 0 | 3.580 | 13.10 |
| Fermented by traditional processes (e.g. ikivugoto) | 0 | 1.680 | 6.68 |
| Heat treated | 0 | 0.450 | 3.60 |
| Fermented by industrial processes | 0 | 1.210 | 5.34 |
| Other dairy products | 0 | 0.408 | 2.67 |

## YLD rate (per 100,000 population)

| Food | 2.5% | Mean | 97.5% |
| --- | --- | --- | --- |
| DAIRY | 0 | 0.08100 | 0.3370 |
| Milk from cattle | 0 | 0.07620 | 0.3180 |
| Milk from other animals | 0 | 0.00482 | 0.0437 |
| Consumed raw | 0 | 0.03730 | 0.1660 |
| Fermented by traditional processes (e.g. ikivugoto) | 0 | 0.01760 | 0.0855 |
| Heat treated | 0 | 0.00460 | 0.0377 |
| Fermented by industrial processes | 0 | 0.01260 | 0.0659 |
| Other dairy products | 0 | 0.00417 | 0.0281 |

```

Children under 5 years of age

## Incidence

| Food | 2.5% | Mean | 97.5% |
| --- | --- | --- | --- |
| DAIRY | 0 | 3320 | 17300 |
| Milk from cattle | 0 | 3120 | 16300 |
| Milk from other animals | 0 | 199 | 1770 |
| Consumed raw | 0 | 1520 | 8580 |
| Fermented by traditional processes (e.g. ikivugoto) | 0 | 725 | 4380 |
| Heat treated | 0 | 185 | 1540 |
| Fermented by industrial processes | 0 | 515 | 3170 |
| Other dairy products | 0 | 169 | 1250 |

## Mortality

| Food | 2.5% | Mean | 97.5% |
| --- | --- | --- | --- |
| DAIRY | 0 | 4.860 | 16.30 |
| Milk from cattle | 0 | 4.570 | 15.80 |
| Milk from other animals | 0 | 0.287 | 2.69 |
| Consumed raw | 0 | 2.230 | 8.33 |
| Fermented by traditional processes (e.g. ikivugoto) | 0 | 1.050 | 4.26 |
| Heat treated | 0 | 0.281 | 2.23 |
| Fermented by industrial processes | 0 | 0.753 | 3.34 |
| Other dairy products | 0 | 0.255 | 1.68 |

## DALY

| Food | 2.5% | Mean | 97.5% |
| --- | --- | --- | --- |
| DAIRY | 0 | 440.0 | 1470 |
| Milk from cattle | 0 | 414.0 | 1430 |
| Milk from other animals | 0 | 26.1 | 245 |
| Consumed raw | 0 | 202.0 | 752 |
| Fermented by traditional processes (e.g. ikivugoto) | 0 | 95.0 | 386 |
| Heat treated | 0 | 25.5 | 202 |
| Fermented by industrial processes | 0 | 68.3 | 303 |
| Other dairy products | 0 | 23.1 | 152 |

## YLL

| Food | 2.5% | Mean | 97.5% |
| --- | --- | --- | --- |
| DAIRY | 0 | 435.0 | 1460 |
| Milk from cattle | 0 | 409.0 | 1420 |
| Milk from other animals | 0 | 25.7 | 241 |
| Consumed raw | 0 | 200.0 | 746 |
| Fermented by traditional processes (e.g. ikivugoto) | 0 | 93.9 | 382 |
| Heat treated | 0 | 25.2 | 200 |
| Fermented by industrial processes | 0 | 67.5 | 300 |
| Other dairy products | 0 | 22.8 | 151 |

## YLD

| Food | 2.5% | Mean | 97.5% |
| --- | --- | --- | --- |
| DAIRY | 0 | 5.090 | 26.30 |
| Milk from cattle | 0 | 4.780 | 25.10 |
| Milk from other animals | 0 | 0.306 | 2.69 |
| Consumed raw | 0 | 2.340 | 13.00 |
| Fermented by traditional processes (e.g. ikivugoto) | 0 | 1.110 | 6.79 |
| Heat treated | 0 | 0.285 | 2.38 |
| Fermented by industrial processes | 0 | 0.792 | 4.82 |
| Other dairy products | 0 | 0.257 | 1.90 |

## Incidence rate (per 100,000 population)

| Food | 2.5% | Mean | 97.5% |
| --- | --- | --- | --- |
| DAIRY | 0 | 186.00 | 969.0 |
| Milk from cattle | 0 | 175.00 | 914.0 |
| Milk from other animals | 0 | 11.20 | 99.1 |
| Consumed raw | 0 | 85.30 | 480.0 |
| Fermented by traditional processes (e.g. ikivugoto) | 0 | 40.60 | 245.0 |
| Heat treated | 0 | 10.40 | 86.2 |
| Fermented by industrial processes | 0 | 28.90 | 178.0 |
| Other dairy products | 0 | 9.44 | 70.0 |

## Mortality rate (per 100,000 population)

| Food | 2.5% | Mean | 97.5% |
| --- | --- | --- | --- |
| DAIRY | 0 | 0.2720 | 0.9140 |
| Milk from cattle | 0 | 0.2560 | 0.8850 |
| Milk from other animals | 0 | 0.0161 | 0.1510 |
| Consumed raw | 0 | 0.1250 | 0.4660 |
| Fermented by traditional processes (e.g. ikivugoto) | 0 | 0.0587 | 0.2390 |
| Heat treated | 0 | 0.0157 | 0.1250 |
| Fermented by industrial processes | 0 | 0.0422 | 0.1870 |
| Other dairy products | 0 | 0.0143 | 0.0943 |

## DALY rate (per 100,000 population)

| Food | 2.5% | Mean | 97.5% |
| --- | --- | --- | --- |
| DAIRY | 0 | 24.70 | 82.40 |
| Milk from cattle | 0 | 23.20 | 80.10 |
| Milk from other animals | 0 | 1.46 | 13.70 |
| Consumed raw | 0 | 11.30 | 42.10 |
| Fermented by traditional processes (e.g. ikivugoto) | 0 | 5.32 | 21.60 |
| Heat treated | 0 | 1.43 | 11.30 |
| Fermented by industrial processes | 0 | 3.82 | 17.00 |
| Other dairy products | 0 | 1.29 | 8.52 |

## YLL rate (per 100,000 population)

| Food | 2.5% | Mean | 97.5% |
| --- | --- | --- | --- |
| DAIRY | 0 | 24.40 | 81.90 |
| Milk from cattle | 0 | 22.90 | 79.30 |
| Milk from other animals | 0 | 1.44 | 13.50 |
| Consumed raw | 0 | 11.20 | 41.80 |
| Fermented by traditional processes (e.g. ikivugoto) | 0 | 5.26 | 21.40 |
| Heat treated | 0 | 1.41 | 11.20 |
| Fermented by industrial processes | 0 | 3.78 | 16.80 |
| Other dairy products | 0 | 1.28 | 8.45 |

## YLD rate (per 100,000 population)

| Food | 2.5% | Mean | 97.5% |
| --- | --- | --- | --- |
| DAIRY | 0 | 0.2850 | 1.470 |
| Milk from cattle | 0 | 0.2680 | 1.410 |
| Milk from other animals | 0 | 0.0172 | 0.151 |
| Consumed raw | 0 | 0.1310 | 0.729 |
| Fermented by traditional processes (e.g. ikivugoto) | 0 | 0.0623 | 0.380 |
| Heat treated | 0 | 0.0160 | 0.133 |
| Fermented by industrial processes | 0 | 0.0444 | 0.270 |
| Other dairy products | 0 | 0.0144 | 0.107 |

```

Children over 5 years of age and adults

## Incidence

| Food | 2.5% | Mean | 97.5% |
| --- | --- | --- | --- |
| DAIRY | 0 | 4240 | 20300 |
| Milk from cattle | 0 | 3990 | 19400 |
| Milk from other animals | 0 | 248 | 2310 |
| Consumed raw | 0 | 1960 | 9960 |
| Fermented by traditional processes (e.g. ikivugoto) | 0 | 911 | 4690 |
| Heat treated | 0 | 246 | 2080 |
| Fermented by industrial processes | 0 | 654 | 3710 |
| Other dairy products | 0 | 223 | 1590 |

## Mortality

| Food | 2.5% | Mean | 97.5% |
| --- | --- | --- | --- |
| DAIRY | 0 | 6.470 | 21.10 |
| Milk from cattle | 0 | 6.090 | 20.40 |
| Milk from other animals | 0 | 0.385 | 3.61 |
| Consumed raw | 0 | 2.970 | 10.90 |
| Fermented by traditional processes (e.g. ikivugoto) | 0 | 1.400 | 5.58 |
| Heat treated | 0 | 0.373 | 3.03 |
| Fermented by industrial processes | 0 | 1.000 | 4.41 |
| Other dairy products | 0 | 0.338 | 2.15 |

## DALY

| Food | 2.5% | Mean | 97.5% |
| --- | --- | --- | --- |
| DAIRY | 0 | 413.0 | 1340 |
| Milk from cattle | 0 | 388.0 | 1290 |
| Milk from other animals | 0 | 24.5 | 230 |
| Consumed raw | 0 | 190.0 | 687 |
| Fermented by traditional processes (e.g. ikivugoto) | 0 | 89.3 | 354 |
| Heat treated | 0 | 23.8 | 193 |
| Fermented by industrial processes | 0 | 63.8 | 281 |
| Other dairy products | 0 | 21.5 | 137 |

## YLL

| Food | 2.5% | Mean | 97.5% |
| --- | --- | --- | --- |
| DAIRY | 0 | 409.0 | 1330 |
| Milk from cattle | 0 | 385.0 | 1290 |
| Milk from other animals | 0 | 24.3 | 228 |
| Consumed raw | 0 | 188.0 | 681 |
| Fermented by traditional processes (e.g. ikivugoto) | 0 | 88.5 | 351 |
| Heat treated | 0 | 23.6 | 191 |
| Fermented by industrial processes | 0 | 63.2 | 279 |
| Other dairy products | 0 | 21.3 | 136 |

## YLD

| Food | 2.5% | Mean | 97.5% |
| --- | --- | --- | --- |
| DAIRY | 0 | 3.690 | 16.60 |
| Milk from cattle | 0 | 3.480 | 15.80 |
| Milk from other animals | 0 | 0.216 | 2.00 |
| Consumed raw | 0 | 1.700 | 8.21 |
| Fermented by traditional processes (e.g. ikivugoto) | 0 | 0.793 | 3.89 |
| Heat treated | 0 | 0.214 | 1.77 |
| Fermented by industrial processes | 0 | 0.569 | 3.12 |
| Other dairy products | 0 | 0.194 | 1.36 |

## Incidence rate (per 100,000 population)

| Food | 2.5% | Mean | 97.5% |
| --- | --- | --- | --- |
| DAIRY | 0 | 46.90 | 224.0 |
| Milk from cattle | 0 | 44.10 | 214.0 |
| Milk from other animals | 0 | 2.74 | 25.6 |
| Consumed raw | 0 | 21.70 | 110.0 |
| Fermented by traditional processes (e.g. ikivugoto) | 0 | 10.10 | 51.8 |
| Heat treated | 0 | 2.72 | 23.0 |
| Fermented by industrial processes | 0 | 7.22 | 41.0 |
| Other dairy products | 0 | 2.47 | 17.5 |

## Mortality rate (per 100,000 population)

| Food | 2.5% | Mean | 97.5% |
| --- | --- | --- | --- |
| DAIRY | 0 | 0.07150 | 0.2330 |
| Milk from cattle | 0 | 0.06720 | 0.2250 |
| Milk from other animals | 0 | 0.00425 | 0.0399 |
| Consumed raw | 0 | 0.03290 | 0.1200 |
| Fermented by traditional processes (e.g. ikivugoto) | 0 | 0.01550 | 0.0616 |
| Heat treated | 0 | 0.00412 | 0.0335 |
| Fermented by industrial processes | 0 | 0.01110 | 0.0487 |
| Other dairy products | 0 | 0.00373 | 0.0238 |

## DALY rate (per 100,000 population)

| Food | 2.5% | Mean | 97.5% |
| --- | --- | --- | --- |
| DAIRY | 0 | 4.560 | 14.80 |
| Milk from cattle | 0 | 4.290 | 14.30 |
| Milk from other animals | 0 | 0.271 | 2.54 |
| Consumed raw | 0 | 2.100 | 7.59 |
| Fermented by traditional processes (e.g. ikivugoto) | 0 | 0.987 | 3.91 |
| Heat treated | 0 | 0.263 | 2.13 |
| Fermented by industrial processes | 0 | 0.705 | 3.11 |
| Other dairy products | 0 | 0.238 | 1.52 |

## YLL rate (per 100,000 population)

| Food | 2.5% | Mean | 97.5% |
| --- | --- | --- | --- |
| DAIRY | 0 | 4.520 | 14.70 |
| Milk from cattle | 0 | 4.250 | 14.20 |
| Milk from other animals | 0 | 0.269 | 2.52 |
| Consumed raw | 0 | 2.080 | 7.53 |
| Fermented by traditional processes (e.g. ikivugoto) | 0 | 0.978 | 3.88 |
| Heat treated | 0 | 0.261 | 2.11 |
| Fermented by industrial processes | 0 | 0.699 | 3.08 |
| Other dairy products | 0 | 0.236 | 1.50 |

## YLD rate (per 100,000 population)

| Food | 2.5% | Mean | 97.5% |
| --- | --- | --- | --- |
| DAIRY | 0 | 0.04080 | 0.1840 |
| Milk from cattle | 0 | 0.03840 | 0.1750 |
| Milk from other animals | 0 | 0.00238 | 0.0221 |
| Consumed raw | 0 | 0.01880 | 0.0907 |
| Fermented by traditional processes (e.g. ikivugoto) | 0 | 0.00877 | 0.0429 |
| Heat treated | 0 | 0.00236 | 0.0196 |
| Fermented by industrial processes | 0 | 0.00629 | 0.0345 |
| Other dairy products | 0 | 0.00215 | 0.0150 |

```
